# Uncovering atrophy progression pattern and mechanisms in individuals at risk of Alzheimer's disease

**DOI:** 10.1093/braincomms/fcaf099

**Published:** 2025-03-04

**Authors:** Christina Tremblay, Shady Rahayel, Alexandre Pastor-Bernier, Frédéric St-Onge, Andrew Vo, François Rheault, Véronique Daneault, Filip Morys, Natasha Rajah, Sylvia Villeneuve, Alain Dagher, the PREVENT-AD Research Group, John Breitner, Sylvain Baillet, Pierre Bellec, Véronique Bohbot, Mallar Chakravarty, D Louis Collins, Pierre Etienne, Alan Evans, Serge Gauthier, Rick Hoge, Yasser Ituria-Medina, Gerhard Multhaup, Lisa-Marie Münter, Vasavan Nair, Judes Poirier, Natasha Rajah, Pedro Rosa-Neto, Jean-Paul Soucy, Etienne Vachon-Presseau, Sylvia Villeneuve, Philippe Amouyel, Melissa Appleby, Nicholas Ashton, Gülebru Ayranci, Christophe Bedetti, Jason Brandt, Ann Brinkmalm Westman, Claudio Cuello, Mahsa Dadar, Leslie-Ann Daoust, Samir Das, Marina Dauar-Tedeschi, Louis De Beaumont, Doris Dea, Maxime Descoteaux, Marianne Dufour, Sarah Farzin, Fabiola Ferdinand, Vladimir Fonov, David Fontaine, Guylaine Gagné, Julie Gonneaud, Justin Kat, Christina Kazazian, Anne Labonté, Marie-Elyse Lafaille-Magnan, Marc Lalancette, Jean-Charles Lambert, Jeannie-Marie Leoutsakos, Claude Lepage, Cécile Madjar, David Maillet, Jean-Robert Maltais, Sulantha Mathotaarachchi, Ginette Mayrand, Diane Michaud, Thomas Montine, John Morris, Véronique Pagé, Tharick Pascoal, Sandra Peillieux, Mirela Petkova, Pierre Rioux, Mark Sager, Eunice Farah Saint-Fort, Mélissa Savard, Reisa Sperling, Shirin Tabrizi, Pierre Tariot, Eduard Teigner, Ronald Thomas, Paule-Joanne Toussaint, Miranda Tuwaig, Vinod Venugopalan, Sander Verfaillie, Jacob Vogel, Karen Wan, Seqian Wang, Elsa Yu, Alzheimer’s Disease Neuroimaging Initiative (ADNI), R C Petersen, P S Aisen, L A Beckett, M C Donohue, A C Gamst, D J Harvey, C R Jack, W J Jagust, L M Shaw, A W Toga, J Q Trojanowski, M W Weiner

**Affiliations:** Centre for Advanced Research in Sleep Medicine, Hôpital du Sacré-Cœur de Montréal, Montreal, QC, Canada, H4J 1C5; Montreal Neurological Institute, Department of Neurology and Neurosurgery, McGill University, Montreal, QC, Canada, H3A 2B4; Centre for Advanced Research in Sleep Medicine, Hôpital du Sacré-Cœur de Montréal, Montreal, QC, Canada, H4J 1C5; Department of Medicine, University of Montreal, Montreal, QC, Canada H3C 3J7; Centre for Advanced Research in Sleep Medicine, Hôpital du Sacré-Cœur de Montréal, Montreal, QC, Canada, H4J 1C5; Montreal Neurological Institute, Department of Neurology and Neurosurgery, McGill University, Montreal, QC, Canada, H3A 2B4; Brain Imaging Centre, Douglas Institute Research Centre, Montreal, QC, Canada, H4H 1R3; Integrated Program in Neurosciences, Faculty of Medicine, McGill University, Montreal, QC, Canada, H3G 2M1; Montreal Neurological Institute, Department of Neurology and Neurosurgery, McGill University, Montreal, QC, Canada, H3A 2B4; Sherbrooke Connectivity Imaging Lab (SCIL), Université de Sherbrooke, Sherbrooke, QC, Canada, J1K 0A5; Centre for Advanced Research in Sleep Medicine, Hôpital du Sacré-Cœur de Montréal, Montreal, QC, Canada, H4J 1C5; Montreal Neurological Institute, Department of Neurology and Neurosurgery, McGill University, Montreal, QC, Canada, H3A 2B4; Department of Psychology, Toronto Metropolitan University, Toronto, ON, Canada, M5B 2K3; Brain Imaging Centre, Douglas Institute Research Centre, Montreal, QC, Canada, H4H 1R3; Montreal Neurological Institute, Department of Neurology and Neurosurgery, McGill University, Montreal, QC, Canada, H3A 2B4; Brain Imaging Centre, Douglas Institute Research Centre, Montreal, QC, Canada, H4H 1R3

**Keywords:** Alzheimer’s disease, brain atrophy, structural connectivity, protein propagation, serotonin receptor

## Abstract

Alzheimer's disease is associated with pre-symptomatic changes in brain morphometry and accumulation of abnormal tau and amyloid-beta pathology. Studying the development of brain changes prior to symptoms onset may lead to early diagnostic biomarkers and a better understanding of Alzheimer's disease pathophysiology. Alzheimer's disease pathology is thought to arise from a combination of protein accumulation and spreading via neural connections, but how these processes influence brain atrophy progression in the pre-symptomatic phases remains unclear. Individuals with a family history of Alzheimer's disease (FHAD) have an elevated risk of Alzheimer's disease, providing an opportunity to study the pre-symptomatic phase. Here, we used structural MRI from three databases (Alzheimer's Disease Neuroimaging Initiative, Pre-symptomatic Evaluation of Experimental or Novel Treatments for Alzheimer Disease and Montreal Adult Lifespan Study) to map atrophy progression in FHAD and Alzheimer's disease and assess the constraining effects of structural connectivity on atrophy progression. Cross-sectional and longitudinal data up to 4 years were used to perform atrophy progression analysis in FHAD and Alzheimer's disease compared with controls. PET radiotracers were also used to quantify the distribution of abnormal tau and amyloid-beta protein isoforms at baseline. We first derived cortical atrophy progression maps using deformation-based morphometry from 153 FHAD, 156 Alzheimer's disease and 116 controls with similar age, education and sex at baseline. We next examined the spatial relationship between atrophy progression and spatial patterns of tau aggregates and amyloid-beta plaques deposition, structural connectivity and neurotransmitter receptor and transporter distributions. Our results show that there were similar patterns of atrophy progression in FHAD and Alzheimer's disease, notably in the cingulate, temporal and parietal cortices, with more widespread and severe atrophy in Alzheimer's disease. Both tau and amyloid-beta pathology tended to accumulate in regions that were structurally connected in FHAD and Alzheimer's disease. The pattern of atrophy and its progression also aligned with existing structural connectivity in FHAD. In Alzheimer's disease, our findings suggest that atrophy progression results from pathology propagation that occurred earlier, on a previously intact connectome. Moreover, a relationship was found between serotonin receptor spatial distribution and atrophy progression in Alzheimer's disease. The current study demonstrates that regions showing atrophy progression in FHAD and Alzheimer's disease present with specific connectivity and cellular characteristics, uncovering some of the mechanisms involved in pre-clinical and clinical neurodegeneration.

## Teaser

Tremblay *et al*. report similar, but less severe, pattern of atrophy progression in the brain of individuals with a family history of Alzheimer’s disease and Alzheimer's disease. This pattern follows brain connections and is related to the accumulation of two proteins implicated in Alzheimer's disease pathology, tau and beta-amyloid.

## Graphical Abstract

**Graphical Abstract:**
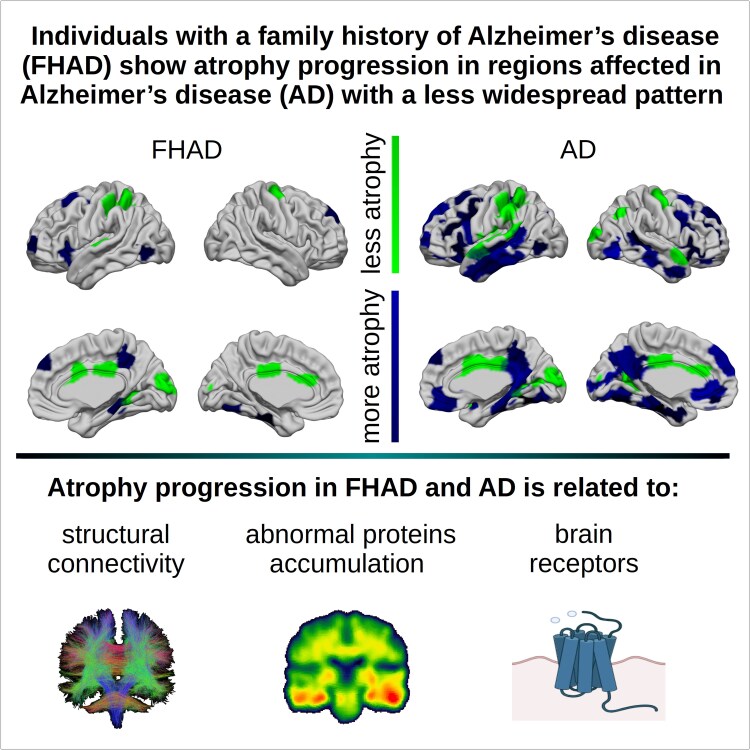

## Introduction

In sporadic Alzheimer's disease, family history stands as the second strongest risk determinant, surpassed only by advanced age.^1^⁠ A meta-analysis revealed a 3.5-fold increase in Alzheimer's disease susceptibility among healthy individuals with a family history of Alzheimer's disease (FHAD), defined as having at least one first-degree relative (a parent or sibling) diagnosed with Alzheimer's disease.^2^⁠ One of the most well-established genetic risk factors is the presence of one or two e4 alleles of the apolipoprotein E gene (*APOe4*).^3^⁠ However, the aetiology of Alzheimer's disease extends beyond a single genetic locus to influence Alzheimer's disease risk as well as brain structure and function.^4^⁠ Family history as a risk factor may encapsulate genetic susceptibilities, as well as other factors that might be transmissible across generations.^5^⁠ Investigating how brain structures are affected in healthy individuals with FHAD could offer insights into the mechanisms underlying the onset of atrophy in sporadic Alzheimer's disease or its alleviation.

Alzheimer's disease pathology is associated with brain tissue loss across various regions, notably the temporal lobe, frontoparietal (FP) and parieto-occipital regions and the hippocampus.^6,7^⁠ While hippocampal atrophy is an early and prominent feature in sporadic Alzheimer's disease,^8,9^⁠ it is not unique to this disease, as it can also occur in conditions such as vascular dementia, semantic dementia and Parkinson's disease with dementia.^10^⁠ Therefore, cortical atrophy patterns may offer a more specific marker for tracking Alzheimer's disease progression, especially in its pre-clinical stages.^10^⁠ In addition to grey matter atrophy, impaired white matter integrity has been observed in Alzheimer's disease. Diffusion-weighted MRI (DW-MRI) enables the construction of white matter tracts through tractography by measuring the diffusion of water molecules along axons.^11^ In sporadic Alzheimer's disease, widespread reductions in fractional anisotropy, generally indicating compromised white matter integrity,^12^ decreased fibre density^13^ and altered structural connectivity, reflected by reduced connection density between regions^14^ have been reported. Alterations in structural connectivity might be present even in the pre-clinical stages of Alzheimer's disease and could influence the propagation of tau aggregates and amyloid-beta (Aβ) depositions, leading to specific atrophy patterns.

While these grey and white matter alterations are commonly observed in Alzheimer's disease, brain structural changes in people with FHAD without overt dementia warrant further investigation. Whole-brain grey matter atrophy^15^ and local atrophy in the precuneus and insula^16^ have been reported, alongside white matter damage (lower fractional anisotropy) in regions also affected in Alzheimer's disease, including the cingulum and uncinate fasciculus.^17,18^⁠ White matter alterations in FHAD have also been associated with higher tau aggregates and Aβ depositions.^18^⁠ Previous findings showed that tau spreads through brain structural connectivity both in normal ageing and Alzheimer's disease and that this propagation is accelerated by Aβ plaques.^19^ However, these studies have not explored how the inter-play among atrophy progression, structural connectivity and pathological protein accumulation specifically manifests in FHAD, leaving a critical gap in understanding the potential early Alzheimer's disease–related pathological mechanisms.

Alterations in neurochemical systems (such as serotonin, glutamate, acetylcholine, histamine, dopamine and norepinephrine) are well-known in sporadic Alzheimer's disease and may increase the vulnerability to atrophy and Alzheimer's disease–related symptoms.^20-22^⁠ Notably, serotonin plays an important role in cognitive functions and memory.^23^ Its depletion has been related to neurodegeneration and depression in Alzheimer's disease.^20^ Excessive glutamate release can also lead to neurotoxicity and contribute to neural degeneration.^20^ In addition, loss of acetylcholine-producing neurons can lead to cognitive decline, and histaminergic dysregulation can contribute to brain inflammation.^20^ Also, dopamine receptors reduction has been associated with cognitive deficit severity in Alzheimer's disease,^21^ while norepinephrine reduction has been linked with neurotoxic pro-inflammatory conditions and reduces Aβ clearance.^22^ However, the specific mechanisms leading to the pattern of atrophy progression in pre-clinical Alzheimer's disease stages and the specific involvement of these neurotransmitter systems are still a matter of debate. Notably, the specific neurotransmitter systems interacting in FHAD to influence atrophy progression remain unknown, highlighting a critical knowledge gap that this study aims to address.

Brain atrophy in Alzheimer's disease might be influenced by multiple factors, including accumulation of tau aggregates and Aβ plaques, reduced structural connectivity and changes in neurotransmitter distribution. We hypothesize that these factors will similarly influence the trajectory of brain atrophy progression in FHAD, but to a milder extent. The primary aim of this study is to elucidate the features underlying cortical atrophy progression in FHAD and sporadic Alzheimer's disease using healthy controls (HCs) as a baseline to distinguish familial risk–related changes from clinical disease-related changes. By elucidating these relationships, we could identify important targets for early disease detection and intervention. We applied deformation-based morphometry (DBM) to quantify cortical atrophy progression in both groups and tractography algorithms to assess whether structural connectivity exerts a constraining effect on atrophy progression, and on the distribution of tau aggregates, and Aβ deposition in each group. Using PET scans, we investigated whether the atrophy progression patterns significantly overlapped with the spatial distributions of tau aggregates and Aβ deposition. Finally, we studied whether atrophy progression patterns occurred within regions expressing specific types of neurochemical receptors and transporters. By integrating these multi-modal imaging approaches, we described how structural and neurochemical factors contribute to atrophy progression in FHAD.

## Materials and methods

### Datasets

Neuroimaging and clinical data for individuals with FHAD, Alzheimer's disease and cognitively unimpaired HC without an FHAD were acquired from three databases: the Pre-symptomatic Evaluation of Experimental or Novel Treatments for Alzheimer's Disease (PREVENT-AD),^24^ The Alzheimer's Disease Neuroimaging Initiative (ADNI),^25^⁠ and the Montreal Adult Lifespan Study (MALS).^26^⁠ PREVENT-AD is a longitudinal study of cognitively normal older adults who have a parent or at least one sibling diagnosed with Alzheimer's disease (FHAD) (see ‘Inclusion criteria: PREVENT-AD’ in the Supplementary material). ADNI's primary goal has been to test whether serial MRI, PET, other biological markers and clinical and neuropsychological assessment can be combined to measure the progression of mild cognitive impairment (MCI) and early Alzheimer's disease (www.adni-info.org). The inclusion criteria in ADNI are described in the Supplementary material. Participants from ADNI (adni.loni.usc.edu) were assigned to one of three groups for our analysis: HC, FHAD or Alzheimer's disease. In the MALS database, participants were healthy adults (age range: 19–76 years) with no history of neurological or psychological illness or FHAD.

All participants in the FHAD group were cognitively normal elderly individuals with no current diagnosis of Alzheimer's disease or other neurological disorders. Each had at least one first-degree relative, a parent or sibling, diagnosed with Alzheimer's disease. Participants in this group were not screened for specific mutations in genes associated with autosomal dominant Alzheimer's disease (e.g. APP, PSEN1 and PSEN2) and may include individuals with varying degrees of genetic and environmental risk factors. While these individuals are at higher risk due to their family history, their eventual development of Alzheimer's disease remains largely unknown. Their inclusion aims to explore potential brain structural changes associated with familial risk. The Alzheimer's disease group comprised individuals with sporadic Alzheimer's disease, as defined by ADNI's criteria, irrespective of family history. According to these criteria, ADNI patients in the Alzheimer's disease group must have a Mini Mental State Examination (MMSE) score from 20 to 26, impairments on the delayed recall of 1 paragraph from the Logical Memory II subscale of the Wechsler Memory Scale–Revised, a clinical dementia rating (CDR) score ≥0.5 and met the National Institute of Neurological and Communicative Disorders and Stroke/Alzheimer’s Disease and Related Disorders Association (NINCDS/ADRDA) criteria for probable Alzheimer's disease. The group with Alzheimer's disease includes patients with and without a family history of the disease. Its purpose is to provide a benchmark for understanding typical cortical atrophy patterns associated with sporadic Alzheimer's disease, independent of familial background. The HC group (healthy elderly without an FHAD) was included to distinguish the cortical atrophy patterns of normal ageing from those observed in FHAD and sporadic Alzheimer's disease. In all datasets, participating centres received approval from a local research ethics committee. All the procedures and tests followed these committees’ guidelines. Informed consent was obtained from each participant according to the Declaration of Helsinki.

### Neuroimaging analysis

**Figure 1:**
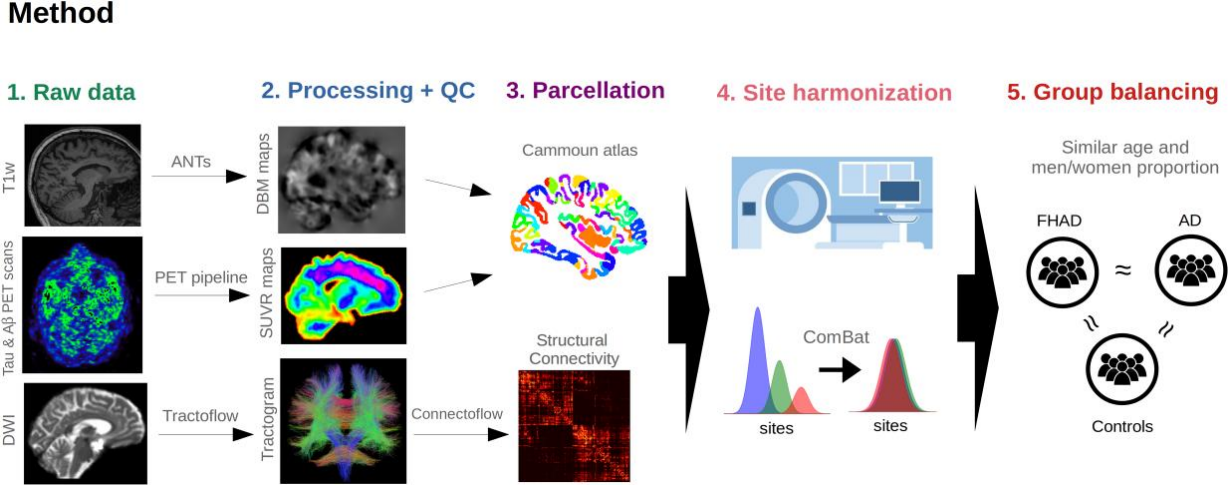
**Five main steps of the method for the three modalities used in this study.** The data were first acquired from three different databases (Step 1). Then, all neuroimaging data were processed using different software specific to each modality, followed by QC (Step 2). Subsequently, each brain map was parcellated using the Cammoun atlas with 448 cortical regions (Step 3), and the site effect was regressed out using the ComBat software (Step 4). Last, we ensured that each group [participants with an FHAD or with Alzheimer's disease and HCs (when applicable)] had a similar age and men/women proportion at baseline (Step 5).

We employed T1-weighted MRI to quantify brain atrophy progression, PET with tau and Aβ radiotracers for mapping Alzheimer-related pathology and diffusion-weighted imaging (DWI) to quantify structural connectivity in FHAD and Alzheimer's disease. Figure 1 provides a summary of the main methodological steps for each of these three modalities.

### Brain atrophy progression

#### Data acquisition

To create cortical atrophy progression maps for individuals with FHAD, Alzheimer's disease and HC, structural MRI data were downloaded from PREVENT-AD (internal database), ADNI and MALS in August 2021. Adults (>55 years) with T1-weighted MRI at baseline (from internal PREVENT-AD, ADNI and MALS) and follow-up MRIs over a period of up to 4 years (from internal PREVENT-AD and ADNI) were included in this analysis. All participants were over 55 years (see ‘Inclusion criteria: PREVENT-AD and Inclusion criteria: ADNI’ in Supplementary material). The acquisition parameters for the PREVENT-AD and MALS datasets were the same and are described in the study by Tremblay-Mercier *et al*.^25^ All MRI images from these databases were acquired on a Magnetom Tim Trio 3 Tesla (Siemens) scanner at the Douglas Mental Health University Institute (Montreal, QC, Canada). T1-weighted MRI from ADNI, acquired on 3 and 1.5 Tesla scanners, were included (protocol described in the study by Jack *et al*.^26^). ADNI has implemented standardized neuroimaging protocols across its participating centres to ensure consistency and reliability in data acquisition. ADNI's approach includes the development of specific MRI protocols tailored for various scanner platforms from different manufacturers. These protocols were optimized and validated through a series of evaluations and a reduced scale clinical trial to ensure they met the study's requirements.^26^ Moreover, to maintain uniformity across sites, ADNI employs post-acquisition corrections and phantom-based monitoring of all scanners. The number of participants at each time point and main steps of the method are detailed in Table 1 (see Supplementary Table 1 for the number of subjects in each database separately).

**Table 1:** Number of subjects at each main step of the method for the patients with Alzheimer's disease and the individuals with an FHAD

Data	Group	Database	Raw data		Processing + QC		Parcellation	Site effect harmonization		Group balancing	
**Atrophy progression—DBM**											
T1w-MRI	Alzheimer's disease	ADNI	Bl:418	1Y:237	Bl:340	1Y:190	No data removed	No data removed		Bl:156	1Y:96
			2Y:153	3Y:18	2Y:121	3Y:17				2Y:55	3Y:5
	FHAD	ADNI PREVENT-AD	Bl:320	1Y:216	Bl:280	1Y:148	No data removed	No data removed		Bl:153	1Y:126
			2Y:179	3Y:65	2Y:155	3Y:53				2Y:110	3Y:46
			4Y:99		4Y:83					4Y:65	
	HC	ADNI MALS	Bl:153	1Y:216	Bl:131	1Y:55	No data removed	Bl:127	1Y:55	Bl:116	1Y:47
			2Y:70	3Y:48	2Y:56	3Y:39		2Y:56	3Y:39	2Y:48	3Y:33
			4Y:27		4Y:20			4Y:20		4Y:18	
**PET imaging**											
Tau-PET	Alzheimer's disease	ADNI	Bl:64		Bl:62		No data removed	Bl:58		Bl:58	
	FHAD	ADNI PREVENT-AD	Bl:166		Bl:157		No data removed	Bl:146		Bl:96	
Ab-PET	Alzheimer's disease	ADNI	Bl:167		Bl:159		No data removed	Bl:154		Bl:145	
	FHAD	ADNI PREVENT-AD	Bl:221		Bl:219		No data removed	Bl:214		Bl:165	
**Structural connectivity—diffusion tensor imaging**											
DWI-MRI	Alzheimer's disease	ADNI	Bl:116		Bl:91		Bl:90	No data removed		Bl:72	
	FHAD	PREVENT-AD	Bl:304		Bl:277		Bl:274	No data removed		Bl:78	

Bl, baseline.

#### Processing and quality control

DBM was used as a voxel-wise measure of brain atrophy. DBM maps were derived from each participant's T1-weighted MRI image at each time point using the Advanced Normalization Tools Longitudinal Cortical Thickness pipeline [see ‘Deformation-based morphometry (DBM) processing’ in Supplementary material].^27^ Quality control (QC) was done by visual inspection of the resultant DBM maps. In total, 158 subjects with Alzheimer's disease (19%), 40 FHAD subjects (18%) and 67 HC (18%) were excluded mostly due to inaccurate grey matter segmentation (see Table 1 for the remaining number of subjects at each time point).

#### Parcellation, site harmonization and group balancing

DBM maps were parcellated using the Cammoun atlas consisting of 448 cortical regions.^28^ This parcellation was chosen because it provides a higher regional resolution than the Desikan–Killiany (DK) (68 regions) and DK–Tourville (62 regions) atlases, allowing for a more fine-grained assessment of cortical atrophy. Using the Cammoun atlas enabled us to compare multi-modal MRI measures (DBM and PET maps) and neurotransmitter maps. To account for scanning site variability, we used Longitudinal ComBat (LongComBat), a Bayes harmonization method developed and validated for longitudinal brain imaging data.^29^ This statistical harmonization method is specifically designed to correct for scanner-related effects, including differences in magnetic field strength (e.g, notably the differences between 1.5T and 3T scanners in ADNI). LongComBat was applied region wise to regress out site-specific effects (age, sex and group were used as covariates). Four subjects with Alzheimer's disease were excluded because they were the only subjects for their site. To ensure that the three groups were similar in terms of age at baseline, individuals with an FHAD who were under 66 years old and HC and Alzheimer's disease subjects over 84 years of age were removed (mean FHAD: 72.6 years, range: 66–87.8 years; mean Alzheimer's disease: 72.8 years, range: 55.2–83.7 years and HC: 72.6 years, range: 60.0–83.4 years; *P-*value = 0.94). The groups were also similar in terms of education (mean FHAD: 16.1 years, range: 9–20 years; mean Alzheimer's disease: 15.6 years, range: 13–20 years and HC: 16.1 years, range: 7–20 years; *P-*value = 0.15) and sex (FHAD = 56% women versus HC = 63% women: *χ*^2^ = 3.67, *P-*value *=* 0.06; Alzheimer's disease = 51% women versus HC = 63% women: *χ*^2^ = 3.67, *P-*value *=* 0.06). Ultimately, this study included data from 153 subjects with FHAD (85 women), 156 subjects with Alzheimer's disease (80 women) and 116 HC (73 women) at baseline, with follow-up scans for up to 4 years.

#### Participants

Table 2 describes the clinical characteristics of the participants with Alzheimer's disease, FHAD and HC at baseline. Two-tailed *F*-tests with *post hoc* comparisons and Bonferroni–Holm correction were used to compare the groups. Compared with HC, the Montreal Cognitive Assessment (MoCA) score,^30^ corrected for education, was significantly lower in Alzheimer's disease (mean Alzheimer's disease: 16.3 versus HC: 27.1; *P*-value < 0.0001) as well as the MMSE score (mean Alzheimer's disease: 23.1 versus HC: 29.2; *P*-value < 0.0001). Subjects with missing data were removed from the analysis. Baseline MoCA scores were unavailable for 68 Alzheimer's disease, 25 FHAD and 38 HC participants, while baseline MMSE scores were missing for 45 FHAD and 1 HC participants. Additionally, a small amount of data was missing for the APOe4 status: 8 in the Alzheimer's disease group, 2 in the FHAD group and 1 in the HC group. A higher proportion of Alzheimer's disease subjects (70%) compared with HC (26%) carried either one (*P-*value *<* 0.001) or two APOe4 alleles (*P-*value *< 0*.001), while a slightly higher proportion of FHAD subjects carried two APOe4 alleles (FHAD: 8.6% versus HC: 0.9%; *P-*value = 0.005).

**Table 2:** Descriptive statistics for the controls and participants with an FHAD and Alzheimer's disease included in this study

Characteristics	Baseline control	Baseline FHAD	Baseline Alzheimer's disease	*F*-test	FHAD versus control	Alzheimer's disease versus control
	Mean (SD)	Mean (SD)	Mean (SD)	*P*-value adjusted^a^	*P*-value	*P*-value
Number of subjects	116	153	156	NA	NA	NA
Sex (men/women)^b^	43/73	68/85	76/80	NA	0.22	0.06
Age (years)	72.6 (6.4)	72.6 (5.0)	72.8 (6.7)	0.94	NA	NA
Education (years)	15.6 (2.5)	16.1 (2.6)	16.1 (2.2)	0.30	NA	NA
MMSE^c^	29.2 (1.0)	29.1 (1.0)	23.1 (2.2)	**<0.001**	0.68	**<0.001**
MoCA	27.1 (2.9)	26.1(2.4)	16.3(4.7)	**<0.001**	0.06	**<0.001**
*APOE4* (% of subjects with the gene in one allele)^b^	25%	27%	49%	NA	0.72	**<0.001**
*APOE4* (% of subjects with the gene in two alleles)^b^	0.9%	8.6%	21%	NA	**0.005**	**<0.001**

Bold values indicate statistically significant results (*P*-value < 0.05).

NA, not applicable; GDS, Geriatric Depression Scale; SD, standard deviation.

^a^ *P*-value adjusted with Bonferroni–Holm correction.

^b^ *c* ^2^ test.

^c^Data from ADNI cohort: data available for 59% (control) and 70% (FHAD) of the subjects.

#### Statistical analysis

The atrophy progression pattern in FHAD and Alzheimer's disease was investigated using linear mixed models with random intercept and slope (MATLAB, R2020b). Unstructured covariance matrices were used for the random effects. Mixed-effects models were computed to account for the different numbers of follow-up scans at each time point and the dependency of the data. However, the Kruskal–Wallis test indicated no significant difference [H(2) = 2.24, *P-*value = 0.33] in the number of scans between the groups (FHAD, Alzheimer's disease and HC). The atrophy progression (group × age interaction) was compared region wise between the three groups. Group, age, sex, education, body mass index (BMI), *APOe4* status, and *APOe4* × age interaction were used as covariates. *Post hoc* tests were also computed between each group. Secondary analyses were done to further assess the sex effect by adding a group × sex × age interaction, as well as group × sex and sex × age interactions, in the models. False discovery rate (FDR) corrections were applied to control for multiple comparisons in region-wise analyses (448 regions).^31^ In all subsequent analyses, the regional *β*-values of the group × age interaction were used as an estimate of regional atrophy progression in FHAD and Alzheimer's disease. A positive *b*-value indicates greater atrophy progression with age compared with HC, while a negative *b*-value indicates lower atrophy progression. Additionally, *W*-score maps were generated region wise for atrophy at baseline to account for age and sex effects.^32^ A higher *W*-score indicates greater baseline atrophy compared with HC. Pearson's correlations between FHAD and Alzheimer's disease atrophy progression, and baseline atrophy, were also computed. Their significance was tested against spatial null models using the software BrainSmash (1000 spins).^33,34^

#### Atrophy progression in the resting-state networks

To explore the relationship between brain atrophy and cognitive function, the mean atrophy progression was calculated for each of the seven resting-state networks, as defined by Yeo *et al*.^35^ This network-based approach was chosen based on the hypothesis that cognitive impairments may be more closely associated with atrophy in inter-connected networks rather than in isolated regions. The mean atrophy progression in each network was compared among the three groups (FHAD, Alzheimer's disease and HC) with linear mixed models (random intercept and slope), along with *post hoc* tests. Group, age, sex, education, BMI, *APOe4* status and *APOe4 ×* age interaction were used as covariates. No atrophy progression data were missing for this analysis. Partial Spearman's correlations, adjusting for age at baseline, BMI and *APOe4* status, were also performed to assess the associations between cognitive measures (education-corrected MoCA scores) and baseline atrophy (*W*-score) across each resting-state network (*P-*value_FDR_ < 0.05). Subjects with missing data were removed from the correlations. A total of 128 FHAD subjects and 88 Alzheimer's disease subjects were included in the correlation analyses.

### Positron emission topography imaging

#### Data acquisition

To create tau aggregates and Aβ deposition maps at baseline, PET scans from the PREVENT-AD (internal database) and ADNI (unprocessed data) were used for subjects with FHAD (tau: 166 scans; Aβ: 221 scans) and Alzheimer's disease (tau: 64 scans; Aβ: 167 scans) (Table 1). Each subject had one baseline scan for tau and Aβ, as PREVENT-AD had no follow-up scans and ADNI's follow-up scans were limited. For the subjects with FHAD in the PREVENT-AD database, the radiotracers [18F]AV-1451 (flortaucipir) and [18F]NAV4694 were used to quantify the distribution of tau aggregates and Aβ plaques (tau: 120 scans; Aβ: 122 scans). T1-weighted MRI scans were acquired up to 1 year before the PET scans (mean interval: 8.9 ± 4.8 months). PET scans and T1w-MRI acquisition are described in the Supplementary material [see ‘PET acquisition: PREVENT-AD’]. Baseline PET data from the ADNI database were downloaded in January 2023. The radiotracers [18F]AV-1451 (flortaucipir) and [18F]AV-45 (florbetapir) were used to assess tau aggregates and Aβ plaques distribution in subjects with FHAD and Alzheimer's disease. This dataset included 46 tau-PET and 99 Aβ-PET scans for FHAD and 64 tau-PET and 167 Aβ-PET scans for Alzheimer's disease (Table 1). T1-weighted MRI, acquired up to 1 year prior to the PET scans, were downloaded. Acquisition parameters and protocols for the T1w-MRI are described in the study by Jack *et al*.^26^ PET scanners differed slightly by site; more information on PET image acquisition in ADNI is available at http://adni-info.org and in the Supplementary material (‘PET acquisition: ADNI’).

#### Processing and quality control

PET scans from the PREVENT-AD and ADNI databases were then processed with the same standard pipeline (github.com/villeneuvelab/vlpp) and QC (see ‘PET processing: ADNI and PREVENT-AD’ in the Supplementary material). Standardized uptake value ratios (SUVRs) using the inferior cerebellum grey matter for tau-PET scans^36^ and cerebellum grey matter as the reference region for Aβ-PET scans^37^ were computed voxel wise. QC was performed to exclude images with motion-related artefacts that compromised general image quality (two subjects), incorrect reconstructions of PET images (five subjects), incomplete PET field of view or incomplete reference region in PET images (five subjects) or significant misalignment between PET and T1-weighted images, leading to unreliable spatial localization or quantification (nine subjects). No participants were excluded from the PREVENT-AD cohort. From the ADNI database, nine tau (5%) and two Aβ (1%) scans from FHAD subjects, along with two tau (3%) and eight Aβ (5%) scans from Alzheimer's disease subjects, were excluded.

#### Parcellation, site harmonization and group balancing

The T1w-MRI were parcellated into 448 cortical regions with the Cammoun atlas.^28^ These regions were used to extract SUVRs from the PET maps. Then, ComBat was applied to regress out inter-site variability from the PET images with age, sex and group as covariates.^38^ Data from sites with only one subject were excluded: FHAD: tau (*n* = 11) and Aβ (*n* = 5) and Alzheimer's disease: tau (*n* = 4) and Aβ (*n* = 5). To ensure that the average age of the FHAD group was similar to the average age in the atrophy progression analysis, younger FHAD subjects (<66 years) were excluded (tau-PET: *P-*value = 0.67 and Aβ-PET: *P-*value = 0.38). Older subjects with Alzheimer's disease (>89 years) were also excluded (tau-PET: *P-*value = 0.24 and Aβ-PET: *P-*value = 0.16). Despite PREVENT-AD and ADNI using different Aβ radiotracers for FHAD subjects, the Aβ-PET binding distributions were highly correlated between the two databases (*r* = 0.84, *P-*value_spin_ = 0.001). In total, tau imaging from 96 subjects with FHAD and 58 patients with Alzheimer's disease and Aβ imaging from 165 subjects with FHAD and 145 patients with Alzheimer's disease were used to create the PET maps. Linear regression models were used to compare tau-PET and Aβ-PET binding between the Alzheimer's disease and FHAD groups. These models included age at baseline, sex, education, BMI and *APOe4* status as covariates. FDR corrections for multiple comparisons (448 cortical regions) were applied. In addition, Pearson's correlations against spatial null models (1000 spins) were conducted to explore the relationships of baseline atrophy and its progression with baseline tau-PET and Aβ-PET binding distribution.

### Structural connectivity

#### Data acquisition

To develop group-specific structural connectivity matrices for the FHAD and Alzheimer's disease groups, we used DW-MRI data from the PREVENT-AD (*n* = 304) and ADNI (*n* = 116) databases, respectively (Table 1). For the subjects with FHAD, the baseline DWI data were downloaded from the open PREVENT-AD database in February 2023. Additionally, the baseline DWI data for the Alzheimer's disease subjects were downloaded from the ADNI database in February 2023. The DWI acquisition parameters for both datasets are described in the Supplementary material [see ‘Diffusion-weighted MRI (DWI) acquisition: PREVENT-AD and DWI acquisition: ADNI’].

#### Processing and quality control

Tractography and connectomic pipelines were applied to the DWI data to create binary connectivity matrices for the FHAD and Alzheimer's disease group. The TractoFlow Atlas–Based Segmentation pipeline (TractoFlow-ABS: github.com/scilus/tractoflow), with the connectomics profile, was used to create tractography from raw DWI and T1 re-sampled from FreeSurfer v.6.0.^39,40^ The DWI processing is described in detail in the Supplementary material [see ‘DWI processing: Tractography (Tractoflow-ABS)’]. Using dMRIQCpy, we next produced QC files for each main step to remove DWI data and T1w-MRI showing artefacts.^41^ Five subjects with FHAD failed during the Tractoflow-ABS processing were removed after QC, while 22 DWI images from the FHAD group and 25 DWI images from the Alzheimer's disease group were removed after QC.

#### Parcellation, site harmonization and group balancing

We next used Connectoflow v.1.1.0 (github.com/scilus/connectoflow) to build the structural connectome with the Cammoun atlas (448 cortical regions) for the FHAD and Alzheimer's disease groups.^42-44^ The tractograms generated by Tractoflow-ABS were employed in the Connectoflow pipeline. The Convex Optimization Modelling for Microstructure Informed Tractography (COMMIT2) tool was used to assign to each streamline a weight, which was used for removing false-positive connections.^45^ A connectivity matrix was obtained for 274 subjects (3 subjects failed Connectoflow) with FHAD and 90 subjects with Alzheimer's disease (1 subject failed Connectoflow). ComBat was used to remove inter-site variability with age and sex as covariates for the subjects with Alzheimer's disease.^46^ Then, younger subjects with FHAD (<66 years) and older subjects with Alzheimer's disease (>84 years) were removed to ensure a similar age range compared with the subjects in the atrophy progression analysis (FHAD: 66–88 years; Alzheimer's disease: 55–84 years). Finally, 78 FHAD (mean: 70 years, SD: 4 years) and 72 Alzheimer's disease subjects (mean: 73 years, SD: 7 years) were included to generate the group-average structural connectivity matrices.^47^

#### Network spread analysis

According to the network spread hypothesis, pathology spreads along existing connections between regions.^48^ We tested this hypothesis in both FHAD and Alzheimer's disease using the built group-average structural connectivity matrices and an independent matrix from 70 young healthy adults (age = 29 ± 9 years, 43 men).^49^ A similar approach was previously reported to investigate atrophy spreading in isolated REM sleep behaviour disorder,^50^ Parkinson's disease^51,52^ and schizophrenia.^53^ All these studies used a connectivity matrix from young healthy adults^49^ and did not include a connectivity matrix specific to the pathology investigated. In this study, we performed the analysis both with the connectivity matrix from young adults and the group-average connectivity matrices from the Alzheimer's disease and FHAD participants. For the network analysis, we computed Pearson's correlations between the atrophy progression observed in each region of the Cammoun atlas and the average atrophy progression of their structurally connected neighbors. Then, the significance of the correlations was tested against a spatial null model (BrainSMASH, 1000 spins).^33^ Similar network analyses with Pearson's correlations between tau-PET and Aβ-PET binding in a region (SUVR values) and the average measures in the structurally connected regions were also computed to investigate the protein spreading hypothesis.^19^ Finally, Pearson's correlations were computed between each region and its non-structurally connected regions to verify the role of connectivity (*P-*value_FDR_ < 0.05).

### Neurotransmitter receptors and transporters analysis

We next examined if the regional atrophy progression in FHAD and Alzheimer's disease, as well as baseline atrophy in FHAD, Alzheimer's disease and HC, related to the spatial distribution of seven neurotransmitter receptors or transporters was potentially implicated in Alzheimer's disease.^20,21^ Spatial deformation relative to the MNI152-2009c template was used as baseline atrophy measure (DBM maps at baseline). The receptor and transporter distributions include dopamine (D2), norepinephrine, serotonin [5-hydroxytryptamine receptor 1B (5-HT1B) and 5-hydroxytrypamine-6 (5-HT6)], acetylcholine (VAchT), glutamate (mGluR5) and histamine (H3) derived from a meta-analysis of PET studies.^54^ Receptors or transporters associated with PET tracers with more than one reference were *Z*-scored, and a weighted average was calculated. Pearson's correlations were tested against spatial null models using BrainSMASH (1000 spins: *P-*value_FDR_ < 0.05).^33,34^⁠

## Results

### Atrophy progression

**Figure 2:**
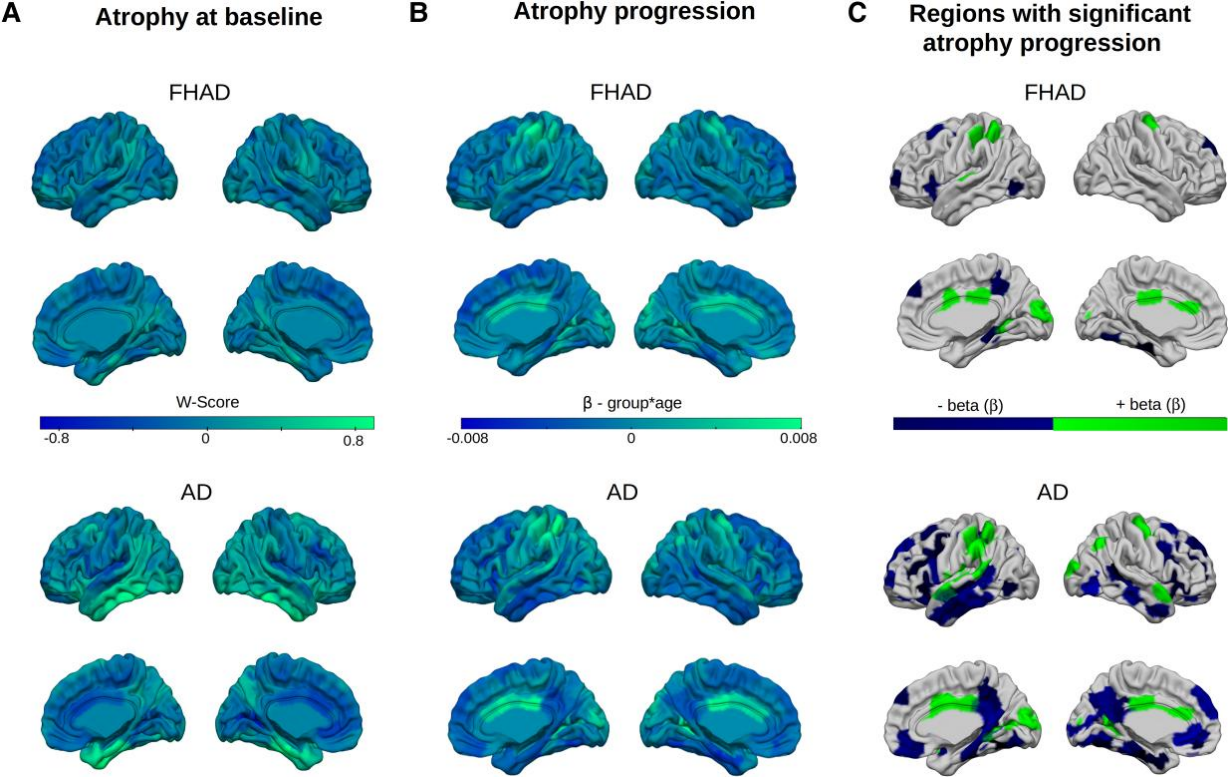
**Brain atrophy progression in individuals with an FHAD and patients with Alzheimer's disease.** (**A**) Baseline atrophy (*W*-scores with age and sex effects in normal aging regressed out) in FHAD and Alzheimer's disease. (**B**) Positive and negative β-values associated with higher and lower atrophy progression in FHAD and Alzheimer's disease compared with HCs. Regions with more baseline atrophy overlap with regions with less atrophy progression. (**C**) Cortical regions showing significant atrophy progression (negative atrophy progression in blue, positive atrophy progression in green) in FHAD and Alzheimer's disease after FDR corrections. Statistical analyses were conducted using linear mixed models with random intercepts and slopes (including group, age, sex, education, BMI, APOe4 status and APOe4 × age interaction as covariates) and *post hoc* tests. The analyses included data from 153 subjects with FHAD, 156 subjects with Alzheimer's disease and 116 HCs.

The DBM maps were used to measure baseline atrophy (Fig. 2A) and atrophy progression in 448 cortical regions in FHAD and Alzheimer's disease (Fig. 2B and C). Suggesting a ceiling effect of atrophy in Alzheimer's disease, more atrophy at baseline (*W*-score) was negatively associated with atrophy progression in this disease (*r* = −0.40, *P-*value_spin_ = 0.001), but not in FHAD (*r* = −0.008, *P-*value_spin_ = 0.87). Significant differences between groups in atrophy progression were observed in 138 cortical regions (*F*-test; *P*-value_FDR_ < 0.05; Supplementary Table 2). In Alzheimer's disease, we found that atrophy progressed significantly with age in 24 regions within the occipital, frontal, temporal, parietal and cingulate cortices, compared with age-expected effects in HC (+β in Supplementary Table 3). In the FHAD group, 10 of these regions and also part of the same cortices showed significant atrophy progression compared with age-expected effects (+β in Supplementary Table 4). Several regions (*n* = 78) showed less atrophy progression in Alzheimer's disease compared with HC, mostly in the temporal and frontal cortices (−β in Supplementary Table 3). In FHAD, 12 of these regions and part of the parietal, temporal, occipital, frontal and limbic cortices also showed less atrophy progression (−β in Supplementary Table 4). These results indicate a slower atrophy progression at older age within specific regions in Alzheimer's disease and FHAD compared with HC. The effect of sex on atrophy progression was investigated, but no significant effect was found for the group × sex × age and group × sex interactions (*P*-value_FDR_ > 0.07), nor for the sex × age interaction (*P-*value_FDR_ > 0.13).

### Comparison between Alzheimer's disease and family history of Alzheimer's disease atrophy patterns

Significant correlations were observed when comparing the baseline atrophy (*W*-score; *r* = 0.33, *P-*value_spin_ = 0.001) and its progression (*r* = 0.75, *P-*value_spin_ = 0.001) between Alzheimer's disease and FHAD, suggesting a similar spatial pattern (Zou's CI: −0.51 to −0.33). However, *post hoc* comparisons between Alzheimer's disease and FHAD revealed that 12 regions exhibited greater atrophy progression (+β value) in Alzheimer's disease and, 80 regions, predominantly located in the temporal, frontal and parietal cortices, demonstrated less atrophy progression with age (−β value) (Supplementary Fig. 1). Overall, these results demonstrate that FHAD subjects show atrophy in regions also affected in Alzheimer's disease, although with a less severe and widespread pattern.

### Atrophy progression in the resting-state networks

**Figure 3:**
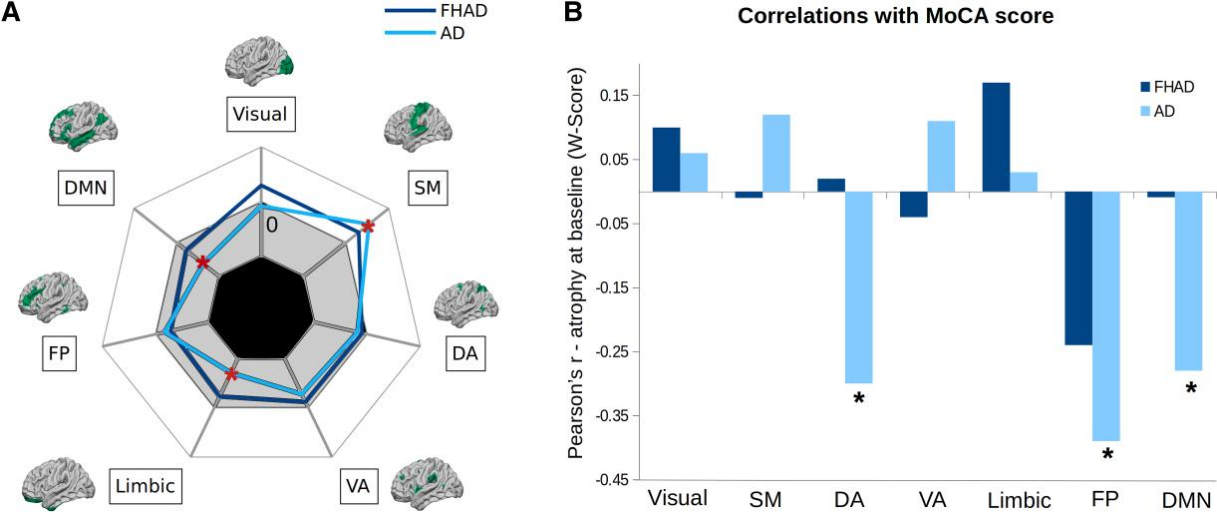
**Brain atrophy progression in the seven resting-state Yeo networks in individuals with an FHAD and patients with Alzheimer's disease.** (**A**) Positive and negative β-values associated with higher [white area: *b*-values range = (0–0.002)] and lower [grey area: *b*-values range = [−0.002 to 0)] atrophy progression in each of the Yeo networks in FHAD and Alzheimer's disease. In Alzheimer's disease, both the limbic network and DMN demonstrated significantly lower atrophy progression compared with HCs. Conversely, the somatosensory network showed an increased rate of atrophy progression. The mean atrophy progression within each network was compared across the three groups (*n*_FHAD_ = 153, *n*_Alzheimer's disease_ = 156 and *n*_HC_ = 116) using linear mixed models with random intercepts and slopes, followed by *post hoc* tests. Covariates included group, age, sex, education, BMI, APOE4 status and the APOE4 × age interaction. The asterisk (*) indicates statistically significant values (*P-*value_FDR_ < 0.05). Regions shown correspond to the areas in each of the Yeo networks. (**B**) Pearson's correlations between average baseline atrophy (*W*-score) in each of the Yeo networks and the MoCA score, which evaluates general cognitive abilities, in FHAD (*n* = 128) and Alzheimer's disease (*n* = 88). Alzheimer's disease participants with higher baseline atrophy in the DA network, FP network and DMN had lower MoCA scores, indicating more cognitive deficits. SM, somatomotor network; VA, ventral attention network.

We next quantified atrophy progression and baseline atrophy in the seven resting-state networks as defined by Yeo *et al*.^35^ There was a significant group effect on the average atrophy progression in the default mode network (DMN, *P-*value_FDR_ = 0.00002), limbic network (*P-*value_FDR_ = 0.002), somatomotor network (*P*-value_FDR_ = 0.008) and ventral attention network (*P-*value_FDR_ = 0.03) (Fig. 3A). The atrophy progression was significantly different in Alzheimer's disease compared with HC in the default mode (β = −0.001, *P-*value_FDR_ = 0.00009), limbic (β = −0.001, *P-*value_FDR_ = 0.006) and somatomotor (β = 0.001, *P-value*_FDR_ = 0.01) networks. However, the atrophy progression was not significant for FHAD compared with HC. These results indicate that the atrophy progression reached a plateau in the default mode and limbic networks (−β value), but it is still progressing (+β value) in the somatomotor network in Alzheimer's disease. Moreover, the results indicate that Alzheimer's disease patients with more baseline atrophy also have more cognitive impairment (lower MoCA scores) in the dorsal attention (DA) (*r* = −0.30, *P-value*_FDR_ = 0.02), FP (*r* = −0.39, *P-value*_FDR_ = 0.002) and default mode (*r* = −0.28, *P-value*_FDR_ = 0.02) networks (Fig. 3B).

### Association with tau aggregates and Aβ deposition

**Figure 4:**
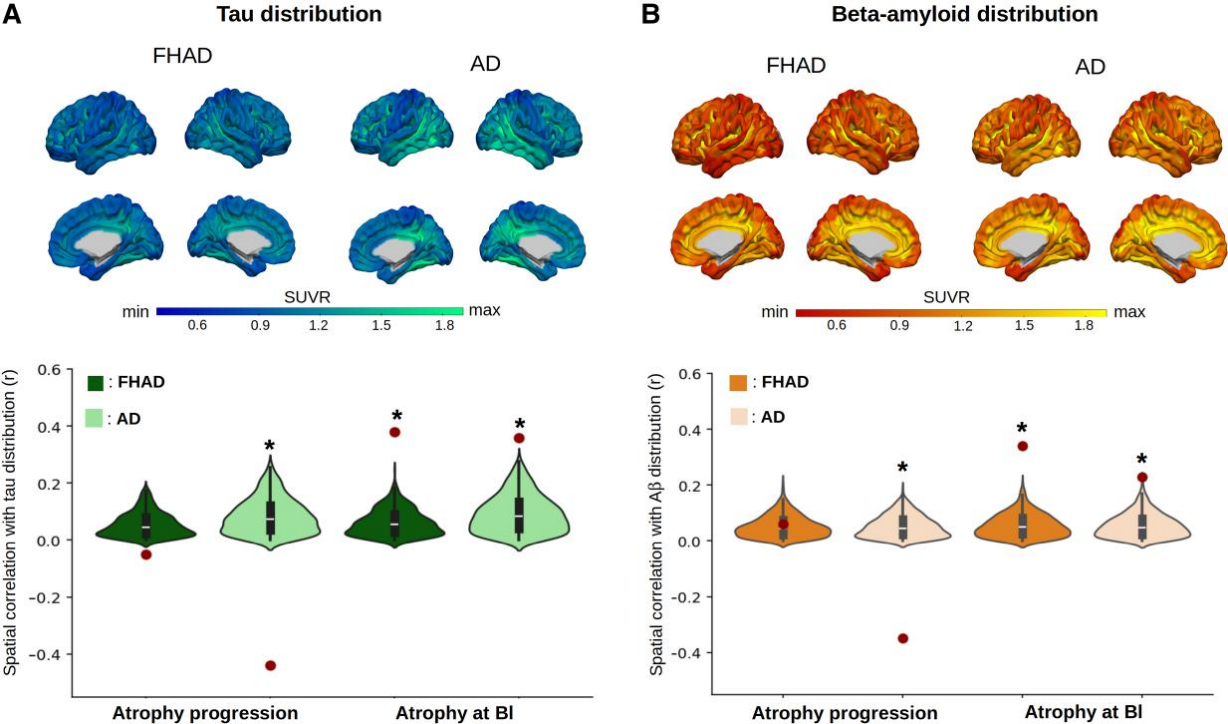
**Relationships between brain atrophy, tau aggregates and beta-amyloid (Aβ) plaques distribution in individuals with an FHAD and Alzheimer's disease.** The patterns of tau aggregates (**A**) and Aβ plaques (**B**) distribution at baseline (Bl) (FHAD: *n*_tau_ = 96, *n*_Aβ_ = 165; Alzheimer's disease: *n*_tau_ = 58, *n*_Aβ_ = 145) were significantly and negatively correlated with the atrophy progression only in Alzheimer's disease (*P-value_spin_ < 0.05). Significant and positive correlations were also found between baseline atrophy and both tau aggregates and Aβ plaques distribution in FHAD and Alzheimer's disease. All correlations (Pearson's *r*, *n*_regions_ = 448) were compared with null coefficient distributions using a model preserving spatial auto-correlation between regions. The dots represent the spatial correlation coefficients for each group, while the asterisks (*) indicate statistically significant values after comparison with a spatial null distribution (1000 spins, *P-*value_spin_ < 0.05). min, minimum; max, maximum.

Tau-PET and Aβ-PET binding were compared between the Alzheimer's disease and FHAD groups. Greater binding were found in Alzheimer's disease compared with FHAD in 358 cortical regions for tau-PET and 335 regions for Aβ-PET (Supplementary Fig. 2). However, both tau-PET and Aβ-PET binding distribution in Alzheimer's disease showed significant spatial overlap with tau-PET (*r* = 0.91, *P-*value_spin_ = 0.001) and Aβ-PET (*r* = 0.94, *P*-value_spin_ = 0.001) binding distribution in FHAD. This suggests that the patterns of regional distributions are similar in the two groups (Fig. 4, upper panel), but that tau aggregates and Aβ deposition are higher in Alzheimer's disease in most cortical regions.

The relationships of baseline atrophy and its progression with baseline tau-PET and Aβ-PET binding distribution patterns were also explored (Fig. 4, lower panel). In Alzheimer's disease, significant correlations were observed between atrophy at baseline and both tau-PET (*r* = 0.36, *P*-value_spin_ = 0.001) and Aβ-PET binding (*r* = 0.23, *P-*value_spin_ = 0.001). Moreover, lower atrophy progression with age was related with more tau-PET (*r* = −0.44, *P-*value_spin_ = 0.001) and Aβ-PET binding (*r* = −0.35, *P-*value_spin_ = 0.001) in this group. In FHAD, significant correlations were found between the patterns of baseline atrophy and both tau-PET (*r* = 0.38, *P-*value_spin_ = 0.001) and Aβ-PET (*r* = 0.34, *P-value*_spin_ = 0.001) binding. However, no significant correlation was observed between the spatial patterns of atrophy progression and tau-PET (*r* = −0.05, *P-*value_spin_ = 0.26) or Aβ-PET (*r* = 0.06, *P-*value_spin_ = 0.18) binding distribution in this group. Comparable associations were observed when analysing only participants with Alzheimer's disease or FHAD who had both PET and MRI data available at baseline (Supplementary Fig. 3). In sum, both tau aggregates and Aβ deposition distribution were related with the atrophy pattern in Alzheimer's disease and FHAD, but not with the subsequent atrophy progression in FHAD.

In addition, the strength of the correlations was compared between tau-atrophy and Aβ-atrophy and between groups. Tau-PET and Aβ-PET correlations with atrophy progression were similar in both groups (Alzheimer's disease: *r*_tau-PET_ = −0.44 versus *r*_Aβ-PET_ = −0.35, *P-*value = 0.11; FHAD: *r*_tau-PET_ = −0.05 versus *r*_Aβ-PET_ = 0.06, *P-*value = 0.10), while tau-PET had a higher correlation with atrophy at baseline, compared with Aβ-PET, only in Alzheimer's disease (Alzheimer's disease: *r*_tau-PET_ = 0.36 versus *r*_Aβ-PET_ = 0.23, *P-*value = 0.03; FHAD: *r*_tau-PET_ = 0.38 versus *r*_Aβ-PET_ = 0.34, *P-*value = 0.49). When comparing Alzheimer's disease with FHAD, a stronger correlation of tau-PET and Aβ-PET with atrophy progression was observed in Alzheimer's disease (tau-PET : *r*_Alzheimer's disease_ = −0.44 versus *r*_FHAD_ = −0.05, *P-*value < 0.0001; Aβ-PET: *r*_Alzheimer's disease_ = −0.35 versus *r*_FHAD_ = 0.06, *P-*value < 0.0001), but no difference was observed with baseline atrophy (tau-PET : *r*_Alzheimer's disease_ = 0.36 versus *r*_FHAD_ = 0.38, *P-*value = 0.73; Aβ-PET: *r*_Alzheimer's disease_ = 0.23 versus *r*_FHAD_ = 0.34, *P-*value = 0.07). These results highlight group-specific differences and underscore the complex inter-play between pathology and neurodegeneration.

### Association with structural connectivity

**Figure 5:**
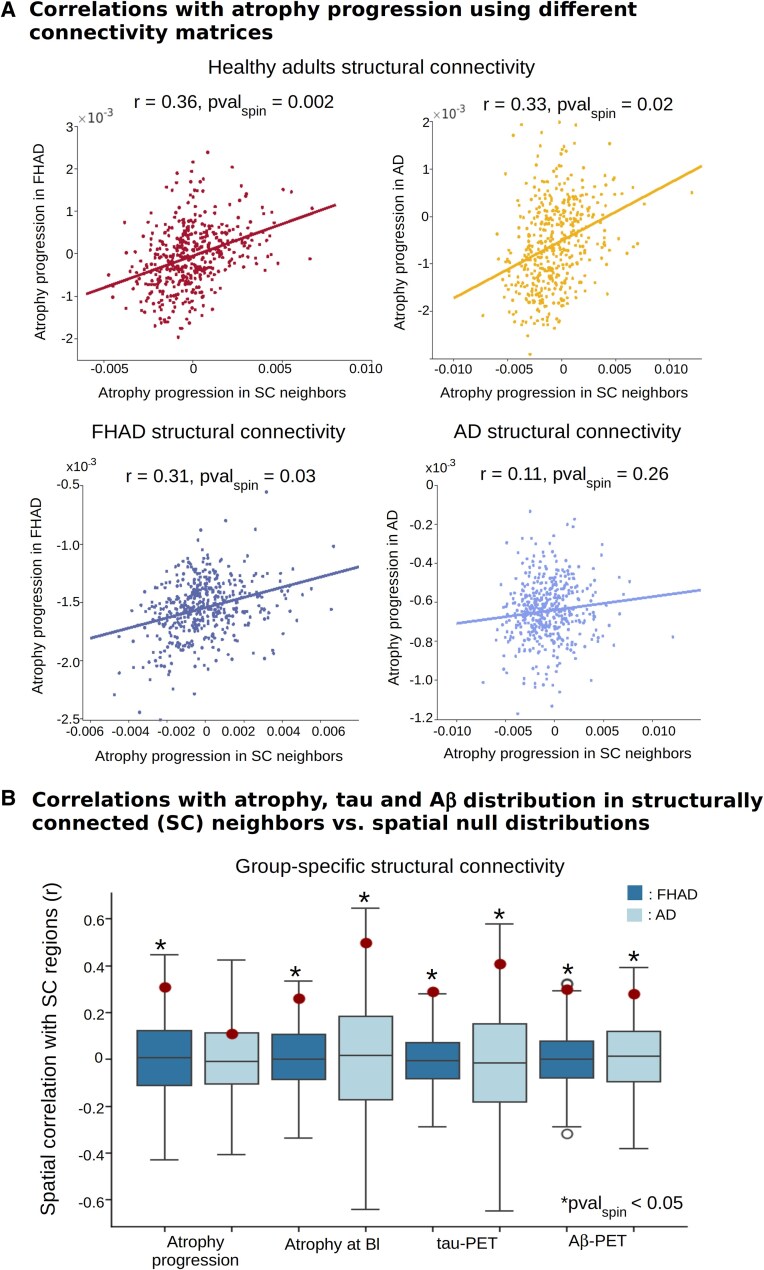
**Relationships with structural connectivity in participants with an FHAD and patients with Alzheimer's disease.** (**A**) Shows the spatial correlations (Pearson's *r*, *n*_regions_ = 448) between cortical atrophy progression in a given region and that in its structurally connected (SC) regions in both FHAD (*n*_Baseline_ = 153) and Alzheimer's disease (*n*_Baseline_ = 156). The upper row depicts correlations using a connectivity matrix from healthy adults (*n*_HC_ = 70), while the bottom row displays correlations using the group-specific connectivity matrices (*n*_FHAD_ = 78, *n*_Alzheimer's disease_ = 72). (**B**) Illustrates the spatial Pearson's correlations (*n*_regions_ = 448) between brain atrophy progression (*n*_FHAD_ = 153, *n*_Alzheimer's disease_ = 156), baseline atrophy (*n*_FHAD_ = 153, *n*_Alzheimer's disease_ = 156), tau aggregates (*n*_FHAD_ = 96, *n*_Alzheimer's disease_ = 58), and beta-amyloid (Aβ) plaques (*n*_FHAD_ = 165, *n*_Alzheimer's disease_ = 145) distribution in a region and those in its structurally connected regions, using group-specific structural connectivity matrices (*n*_FHAD_ = 78, *n*_Alzheimer's disease_ = 72). In FHAD, all the correlations were significant, while in Alzheimer's disease, only the correlation with atrophy progression was not significant. The dots represent the spatial correlation coefficients for each group, while the asterisks (*) indicate statistically significant values after comparison with a spatial null distribution (1000 spins, *P-*value_spin_ < 0.05). Bl, baseline; *P-*val_spin_: *P*-value after spin test with spatial auto-correlation.

We next investigated whether the distribution of cortical atrophy and its progression, as well as tau aggregates or Aβ deposition distributions, could be explained by a process propagating via brain connections (Fig. 5). If so, a region's atrophy, atrophy progression or tau aggregates or Aβ deposition should correlate with the same measure in its structurally connected neighbours. We used three connectomes for this analysis: a connectivity matrix from healthy adults and structural connectivity matrices from the subjects with FHAD and Alzheimer's disease. This approach allowed us to compare the role of connectivity prior to the development of pathology and at different stages of the neuropathological process.

In FHAD, using a healthy connectome, we found that the higher the baseline atrophy and its progression, as well as the higher tau-PET and Aβ-PET binding in a region, the higher the baseline atrophy (*r* = 0.44, *P-*value_spin_ = 0.001), atrophy progression (*r* = 0.36, *P-*value_spin_ = 0.02), tau-PET (*r* = 0.24, *P-*value_spin_ = 0.009) and Aβ-PET binding (*r* = 0.29, *P*-value_spin_ = 0.003) in regions that are structurally connected (Supplementary Table 5). In contrast, baseline atrophy, its progression, tau-PET and Aβ-PET binding were lower in regions that were not structurally connected (baseline atrophy: *r* = −0.50, *P-*value_spin_ = 0.001; atrophy progression: *r* = −0.36, *P-*value_spin_ = 0.001; tau: *r* = −0.34, *P-*value_spin_ = 0.001 and Aβ: *r* = −0.39, *P-*value_spin_ = 0.001). Similar results were obtained using the structural connectivity matrix from the FHAD participants (Fig. 5A, left upper row; Supplementary Table 6). These results suggest that in FHAD, distributions of atrophy, tau aggregates and Aβ deposition all depend on structural connections in a similar manner.

In Alzheimer's disease, using an intact connectivity matrix, a significant correlation was found between baseline atrophy in each region and that in their connected neighbours (*r* = 0.66, *P-*value_spin_ = 0.001). Significant correlations were also observed with atrophy progression (*r* = 0.33, *P*-value_spin_ = 0.02), tau aggregates (*r* = 0.49, *P-*value_spin_ = 0.001) and Aβ deposition (*r* = 0.21, *P-*value_spin_ = 0.004) (Fig. 5A, right upper row; Supplementary Table 5). Additionally, negative correlations were noted with the same measures when using the non-structurally connected neighbours (baseline atrophy: *r* = −0.67, *P-*value_spin_ = 0.001; atrophy progression: *r* = −0.43, *P-*value_spin_ = 0.001; tau: *r* = −0.36, *P-*value_spin_ = 0.001 and Aβ: *r* = −0.32, *P-*value_spin_ = 0.001). Using the structural connectivity matrix from Alzheimer's disease, a significant correlation was observed with baseline atrophy (*r* = 0.50, *P-*value_spin_ = 0.001). However, no significant correlations were found in Alzheimer's disease between the atrophy progression in each region and that in their connected neighbours (*r* = 0.11, *P-*value_spin_ = 0.26) (Fig. 5A, right bottom row). Similarly, the correlation with atrophy progression in the non-structurally connected neighbors was not significant (*r* = −0.16, *P-*value_spin_ = 0.18). In addition, significant correlations were observed with tau-PET (*r* = 0.41, *P-*value_spin_ = 0.03) and Aβ-PET (*r* = 0.28, *P-*value_spin_ = 0.02) binding in a region and the average binding in their structurally connected neighbors (Fig. 5B; Supplementary Table 6). These findings support that in Alzheimer's disease, tau aggregates and Aβ deposition accumulate following the Alzheimer's disease–specific structural connectome, whereas most of the atrophy progression rather depends on the healthy connectome before Alzheimer's disease–related damages occurred.

### Relationships with receptor and transporter distributions

**Figure 6:**
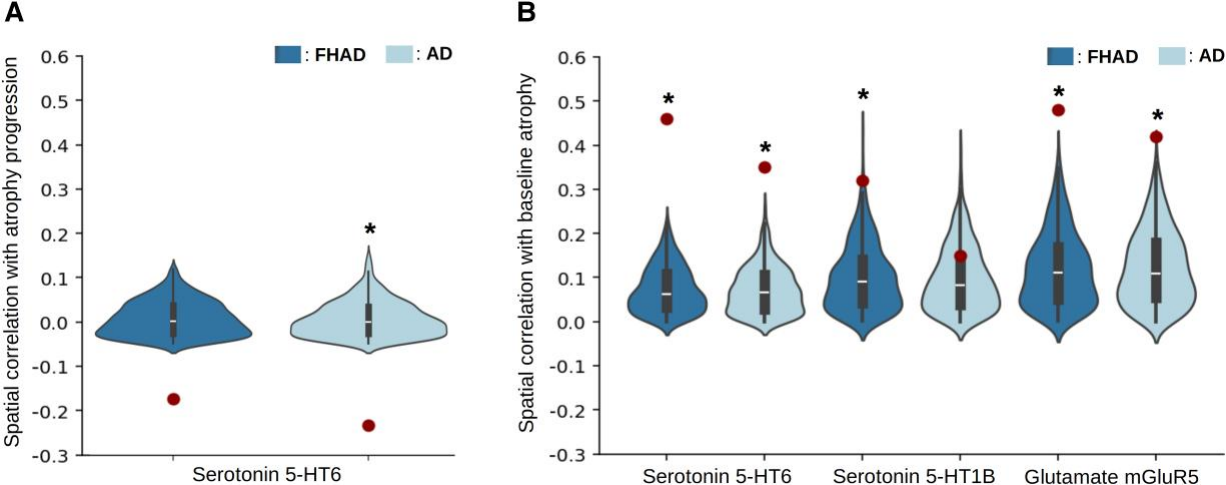
**Serotonin and glutamate receptor distribution related to brain atrophy in individuals with an FHAD and Alzheimer's disease.** (**A**) The spatial distribution of the serotonin 5-HT6 receptor (*n* = 30) in cortical regions (*n* = 448) was negatively correlated with atrophy progression in both FHAD (*n*_Baseline_ = 153) and Alzheimer's disease (*n*_Baseline_ = 156) but was only significant in Alzheimer's disease after comparison with a spatial null distribution and FDR correction. (**B**) Positive and significant correlations were observed between the serotonin 5-HT6 receptor distribution (*n* = 30) and baseline atrophy (*n*_FHAD_ = 153, *n*_Alzheimer's disease_ = 156) in both groups, suggesting that the negative correlations most likely reflect a ceiling effect. In addition, the distribution of the serotonin 5-HT1B receptor (*n* = 88) was significantly correlated with baseline atrophy, but only in FHAD. Finally, significant correlations were observed between the metabotropic glutamate receptor 5 (mGluR5) distribution (*n* = 123) and baseline atrophy (*n*_FHAD_ = 153, *n*_Alzheimer's disease_ = 156) in both groups. All correlations were spatial Pearson's correlations performed across 448 cortical regions. The dots represent the spatial correlation coefficients for each group, while the asterisks (*) indicate statistically significant values after comparison with a spatial null distribution and FDR correction (1000 spins, *P-*value_spin-FDR_ < 0.05).

Since regional factors may also influence the atrophy progression pattern, we examined the spatial correlations between this pattern and the distributions of seven neurotransmitter receptors and transporters (Fig. 6). A negative correlation between the distribution of the 5-HT6 receptors and atrophy progression in FHAD was observed (*r* = −0.15, *P-*value_spin_ = 0.04), but did not remain significant after FDR correction (*r* = −0.15, *P-*value_spin-FDR_ = 0.19). A negative correlation was also found between the distribution of these receptors and the atrophy progression pattern in Alzheimer's disease (*r* = −0.22, *P-*value_spin-FDR_ = 0.04). No other significant correlations were observed with atrophy progression in both groups (Supplementary Table 7). The correlations with baseline atrophy in HC, FHAD, and Alzheimer's disease, and the receptor and transporter distributions were also investigated. Regions with more baseline atrophy possessed higher concentrations of serotonin 5-HT6 and glutamate (mGluR5) receptors in HC (5-HT6: *r* = 0.45, *P-*value_spin-FDR_ = 0.004; mGluR5: *r* = 0.44, *P-*value_spin-FDR_ = 0.004), FHAD (5-HT6: *r* = 0.46, *P-*value_spin-FDR_ = 0.004; mGluR5: *r* = 0.48, *P-*value_spin-FDR_ = 0.004) and Alzheimer's disease (5-HT6: *r* = 0.35, *P*-value_spin-FDR_ = 0.004; mGluR5: *r* = 0.42, *P-*value_spin-FDR_ = 0.004). Associations were also observed with the distribution of serotonin 5-HT1B receptors in HC (*r* = 0.33, *P-*value_spin-FDR_ = 0.01) and FHAD (*r* = 0.32, *P-*value_spin-FDR_ = 0.01), but not in Alzheimer's disease (*r* = 0.15, *P-*value_spin-FDR_ = 0.17). No other significant correlation was observed (Supplementary Table 8). Taken together, these results suggest that more atrophy is observed in regions with higher concentration of serotonin 5-HT6 and mGluR5 receptors in all groups, while a similar association with the serotonin 5-HT1B distribution is only observed in HC and FHAD.

## Discussion

Similar patterns of brain atrophy, atrophy progression and tau aggregates and Aβ deposition were found in FHAD and Alzheimer's disease, with Alzheimer's disease having greater severity and extent. Tau-PET and Aβ-PET binding distributions overlapped spatially with baseline atrophy, but not with subsequent atrophy progression. Structural connectivity analyses revealed that both proteins accumulate in connected regions in FHAD and Alzheimer's disease. The atrophy pattern and its progression also aligned with existing connectivity in FHAD. In Alzheimer's disease, however, the atrophy progression was better explained by the healthy connectome than the connectome derived from the Alzheimer's disease patients. Finally, in all three groups, regions with greater baseline atrophy were associated with higher levels of serotonin 5-HT6 and glutamate (mGluR5) receptor distributions. An association with serotonin 5-HT1B receptor distributions was also observed in HC and FHAD.

### The patterns of atrophy progression

The spatial pattern of cortical atrophy progression was similar in FHAD and Alzheimer's disease, although with greater severity and spatial extent in Alzheimer's disease. Both groups exhibited atrophy progression in specific regions of the cingulate, temporal and parietal cortices. This is consistent with studies reporting atrophy in the cingulate cortex in individuals with MCI and Alzheimer's disease^55,56^ and accelerated atrophy rates in the temporal and parietal cortex in Alzheimer's disease^6,7^⁠ and FHAD.^16,57^ Additionally, our data showed lower atrophy progression at older age compared with HC notably in Alzheimer's disease (possibly reflecting a ceiling effect in the detection of ongoing tissue loss), in the precuneus and regions within the temporal, frontal and occipital cortices. This supports previous findings showing that atrophy begins in the parietal and temporo-occipital cortex, and frontal association areas, before spreading to other cortical regions.^58,59^ Lower cortical atrophy progression and cognitive decline at older age have also been observed in individuals with MCI and Alzheimer's disease.^60^ In Alzheimer's disease, regions with higher baseline atrophy also showed less atrophy progression over time, consistent with a ceiling effect. However, no such association was found in FHAD, possibly due to the small number of regions showing decreased atrophy progression compared with HC.

In addition, we found relationships between the baseline atrophy patterns, and both tau aggregates and Aβ deposition distribution in FHAD and Alzheimer's disease. These results support the associations between these abnormal proteins deposition and brain atrophy.^61,62^ However, there was a negative relationship between atrophy progression and, tau aggregates and Aβ deposition distribution which possibly reflects a ceiling effect in Alzheimer's disease. As expected, the FHAD and Alzheimer's disease groups showed similar patterns in tau aggregates and Aβ deposition distribution with greater deposition in Alzheimer's disease.

### Structural connectivity shapes atrophy and protein propagation

Atrophy progression in specific networks may underlie the more pronounced cognitive dysfunction in Alzheimer's disease compared with FHAD. Alzheimer's disease targets distinct networks, notably the DMN and limbic network.^63^ Our study revealed that, while FHAD did not show significant atrophy in these networks, in Alzheimer's disease, the atrophy plateaued in the DMN and limbic network and progressed in the somatomotor network. Moreover, Alzheimer's disease participants with greater baseline atrophy in either the DMN, FP network, or DA network exhibited more pronounced cognitive deficits. This supports findings of altered activation in the DMN, FP network and somatomotor network during cognitive tasks and resting state in Alzheimer's disease.^64,65^⁠

To further evaluate the role of networks and brain connectivity in pathology progression, we tested whether the atrophy patterns were shaped by structural connectivity. In FHAD and Alzheimer's disease, our analysis revealed that connectivity influences the baseline distribution of tau aggregates, Aβ deposition and atrophy regardless of the connectivity matrix used (group-specific or intact). This is consistent with tau and Aβ network propagation observed in Alzheimer's disease.^19,66^ In Alzheimer's disease alone, Aβ deposition was less influenced by connectivity compared with tau aggregates distribution and baseline atrophy. This supports studies, including Franzmeier *et al*.^67^ and Vogel *et al*.,^19^ showing that tau spreads via functional and structural connectivity in Alzheimer's disease. In FHAD, the atrophy progression pattern aligned with both FHAD-specific and intact connectome, while in Alzheimer's disease, no relationship was observed with Alzheimer's disease–specific connectivity. Localized white matter damage in FHAD does not seem sufficient to disrupt the atrophy progression pattern. However, when using an intact connectome, an association with the connected regions was found in Alzheimer's disease. This suggests that most atrophy progression arises from spreading pathology in a previously intact connectome. Consistent with a ceiling effect of atrophy in Alzheimer's disease, the correlation between current connectivity and atrophy progression was weaker than with baseline atrophy. Overall, our findings support the network spread hypothesis,^68^ emphasizing the critical role of structural connectivity in atrophy progression in FHAD and Alzheimer's disease.

Our results align with models of neurodegeneration that view the brain as a dynamic network where pathological proteins progress along structurally connected pathways. Understanding how structural networks contribute to FHAD and Alzheimer's disease can refine existing models and inform targeted interventions that disrupt this spread. The observation that structural connectivity shapes atrophy and protein propagation highlights the critical role of network integrity in the progression of Alzheimer's disease. This suggests that preserving or restoring connectivity in specific networks, such as the DMN and limbic networks, could be a potential therapeutic target. For example, interventions aimed at enhancing synaptic plasticity or protecting white matter integrity could mitigate the spread of pathological proteins and associated atrophy.

Furthermore, the differential impact of structural connectivity on atrophy progression in FHAD versus Alzheimer's disease implies that FHAD may represent a pre-clinical or early stage of neurodegeneration with distinct mechanisms. Indeed, our observations suggest that FHAD likely represents an earlier and milder form of neurodegeneration compared to sporadic Alzheimer's disease, characterized by less severe and more localized pathology. The inclusion criteria for FHAD, which required normal cognitive function and the absence of neurological or psychiatric disorders, may also have selected individuals in pre-clinical stages with milder presentations. In contrast, the more extensive white matter damage and greater structural connectivity disruption observed in sporadic Alzheimer's disease suggest an accelerated neurodegenerative process compared with FHAD and normal ageing. The milder presentation and less pronounced atrophy progression in FHAD could result from differences in the underlying mechanisms driving pathology, as well as the presence of protective genetic factors that counterbalance the increased risk associated with family history. This suggests that FHAD represents a milder neurodegenerative process and may guide future studies exploring resilience factors in FHAD and their potential application in Alzheimer's disease.

### Association with serotonin and glutamate receptors distribution

The spatial distribution of brain atrophy at baseline was associated with higher regional levels of serotonin 5-HT6 and glutamate (mGluR5) receptors distribution in HC, FHAD and Alzheimer's disease. A negative correlation was also detected between atrophy progression and the spatial distribution of serotonin 5-HT6, most likely reflecting a ceiling effect of atrophy detection in Alzheimer's disease. There was also a positive association between baseline atrophy and the distribution of serotonin 5-HT1B receptors that was unique to FHAD and controls. Both 5-HT6 and 5-HT1B receptors play significant roles in cognition, memory and learning.^69-71^ Our findings are in line with these observations, revealing more atrophy in regions with higher 5-HT6 receptor densities in normal aging, FHAD, and Alzheimer's disease, all conditions where different levels of memory decline occur.^72^ The relationships found with 5-HT1B receptor distribution in normal aging and FHAD suggest that cortical regions with greater distribution of these receptors might be more vulnerable to atrophy in these conditions. In addition, elevated glutamate levels have been implicated in neurotoxicity and Alzheimer's disease neurodegeneration.^73,74^ The mGluR5 receptors might contribute to Aβ toxicity through various mechanisms, eventually leading to tau phosphorylation.^75^ The relationships with the mGluR5 receptor distribution found in this study support the role of this neurotransmitter system in neurodegeneration not only in Alzheimer's disease, but also in normal ageing and FHAD. The association between atrophy and receptor distributions, specifically serotonin 5-HT6 and glutamate (mGluR5) receptors, reveals potential molecular targets for therapeutic strategies. For instance, modulating serotonergic and glutamatergic pathways through receptor-specific drugs could address early cortical vulnerabilities.

### Strengths and limitations

This study has several strengths. It includes three demographically similar groups relative to age, sex and education, including a control group. This allows for a comparison between FHAD and Alzheimer's disease in identifying both shared and distinct patterns of atrophy progression and underlying pathological mechanisms. This study also utilized a multi-modal approach (T1-weighted MRI, diffusion MRI, tau-PET and Aβ-PET scans) to cover multiple facets of Alzheimer's disease and FHAD, including atrophy patterns, structural connectivity effects and abnormal proteins deposition. This provides a holistic view of the brain atrophy progression in both FHAD and Alzheimer's disease. In addition, the study extends beyond the commonly studied tau and Aβ proteins by exploring the role of different receptors and transporters in atrophy. This exploration encompasses not only Alzheimer's disease but also FHAD and normal ageing, thereby offering new insights for research in early intervention strategies.

A few limitations should be noted. This study does not account for all potential confounders, such as medication use or lifestyle factors like physical, cognitive and social activities, which might influence pathology progression and introduce more heterogeneity (variance) in our results. To mitigate the effect of these factors, statistical analyses were performed, controlling for age, education, BMI, sex and *APOe4* status. Racial and ethnic demographics were largely unreported, though the sample was likely predominantly Caucasian, limiting generalizability to other groups. Future research should prioritize diverse populations to improve applicability and explore demographic differences in disease mechanisms. It is also possible that individuals with FHAD are more engage in lifestyle behaviours supporting brain health, such as regular exercise, neuroprotective diets and avoidance of harmful substances, potentially contributing to resilience against disease progression. However, these factors were not captured in our dataset, limiting our ability to assess their potential protective effects in FHAD. Additionally, clinical study participants might differ from the general population in socioeconomic status, healthcare access, and cognitive reserve, which may influence disease progression and resilience factors. Future studies should incorporate lifestyle variables to better understand their role in FHAD and Alzheimer's disease atrophy progression and adopt sampling strategies that more accurately reflect population demographics.

In addition, the study relies on one measure of atrophy, DBM and DWI for structural connectivity. The inclusion of cortical thickness and surface area measurements, as well as functional connectivity, could have enriched the findings. However, DBM was selected as it has previously been shown to adequately detect the pattern of atrophy progression in other neurodegenerative diseases or pre-clinical stages with a similar sample size^50,51,76,77^ and may be more sensitive to detect milder differences in atrophy compared to cortical thickness.^78^ This study also focuses on cortical regions, thereby overlooking the role of sub-cortical areas, such as the hippocampus, which are critical in Alzheimer's disease pathology.^79-82^ While cortical atrophy patterns may offer increased specificity for tracking Alzheimer's disease progression, there may be a trade-off in sensitivity compared to hippocampal atrophy. By focusing solely on cortical regions, we might have missed subtle sub-cortical and hippocampal changes that could enhance the sensitivity of our findings. However, the present study focus on cortical atrophy progression, as it has been overlooked in comparison with sub-cortical structures, and emerging evidence suggests that cortical atrophy patterns may be more specific and sensitive markers for monitoring pre-clinical and early stages of Alzheimer's disease.^10^ Future studies could build upon our findings by incorporating multi-modal imaging and extending the analysis to sub-cortical regions, including the hippocampus, to provide a more holistic understanding of the complex inter-play between connectivity, protein deposition and brain atrophy in FHAD.

## Conclusion

This study delves into the pathological mechanisms at play in FHAD, highlighting both unique and shared neurodegenerative mechanisms between FHAD and Alzheimer's disease. While structural connectivity influences both atrophy and protein propagation in FHAD and Alzheimer's disease, the extent of pathology is less severe and widespread in FHAD. This study also revealed that regions with higher levels of serotonergic and glutamatergic receptors may be especially susceptible to degeneration in normal ageing, FHAD and Alzheimer's disease.

## Supplementary Material

fcaf099_Supplementary_Data

## Data Availability

T1w-MRI and PET data used in this article were from the internal PREVENT-AD database (release 6.0) and ADNI dataset (available at https://adni.loni.usc.edu/data-samples/adni-data/#AccessData). The DWI data are available online at https://openpreventad.loris.ca (PREVENT-AD) and at https://adni.loni.usc.edu/data-samples/adni-data/#AccessData (ADNI). All other datasets and software used are available from the sources cited in the ‘Materials and methods’ section. The data (brain maps and connectivity matrices for the FHAD and Alzheimer's disease groups) and code (MATLAB scripts) generated and used in this study are available at https://gitlab.com/christina.science/papers/atrophy-progression-fhad.

## Appendix I

The list of the contributors involved in the PREVENT-AD Research Group is as follows: John Breitner, Sylvain Baillet, Pierre Bellec, Véronique Bohbot, Mallar Chakravarty, D. Louis Collins, Pierre Etienne, Alan Evans, Serge Gauthier, Rick Hoge, Yasser Ituria-Medina, Gerhard Multhaup, Lisa-Marie Münter, Vasavan Nair, Judes Poirier, Natasha Rajah, Pedro Rosa-Neto, Jean-Paul Soucy, Etienne Vachon-Presseau, Sylvia Villeneuve, Philippe Amouyel, Melissa Appleby, Nicholas Ashton, Gülebru Ayranci, Christophe Bedetti, Jason Brandt, Ann Brinkmalm Westman, Claudio Cuello, Mahsa Dadar, Leslie-Ann Daoust, Samir Das, Marina Dauar-Tedeschi, Louis De Beaumont, Doris Dea, Maxime Descoteaux, Marianne Dufour, Sarah Farzin, Fabiola Ferdinand, Vladimir Fonov, David Fontaine, Guylaine Gagné, Julie Gonneaud, Justin Kat, Christina Kazazian, Anne Labonté, Marie-Elyse Lafaille-Magnan, Marc Lalancette, Jean-Charles Lambert, Jeannie-Marie Leoutsakos, Claude Lepage, Cécile Madjar, David Maillet, Jean-Robert Maltais, Sulantha Mathotaarachchi, Ginette Mayrand, Diane Michaud, Thomas Montine, John Morris, Véronique Pagé, Tharick Pascoal, Sandra Peillieux, Mirela Petkova, Pierre Rioux, Mark Sager, Eunice Farah Saint-Fort, Mélissa Savard, Reisa Sperling, Shirin Tabrizi, Pierre Tariot, Eduard Teigner, Ronald Thomas, Paule-Joanne Toussaint, Miranda Tuwaig, Vinod Venugopalan, Sander Verfaillie, Jacob Vogel, Karen Wan, Seqian Wang and Elsa Yu.
