# Supplementary material for: Uncovering atrophy progression pattern and mechanisms in individuals at risk of Alzheimer's disease
Source: Brain Commun. 2025 Mar 4;7(2):fcaf099. doi: 10.1093/braincomms/fcaf099 (PMC11906971; doi:10.1093/braincomms/fcaf099)
Supplement: fcaf099_Supplementary_Data [file fcaf099_supplementary_data.pdf]

Uncovering atrophy progression pattern and mechanisms in individuals at risk of Alzheimer’s disease

Supplemental Information

Inclusion criteria: PREVENT-AD

Participants enrolled in the study were required (1) to be at least 60 years old, or between 55 and 59 if their age was within 15 years of their first-affected relative's age at the onset of dementia, (2) to not have a history of neurological or psychiatric disorders, and (3) to have normal cognitive functions as indicated by a neuropsychological evaluation.

Inclusion criteria: ADNI

Participants with Mini-Mental Status Exam (MMSE) scores from 24 to 30, a normal delayed recall of 1 paragraph from the Logical Memory II subscale of the Wechsler Memory Scale–Revised and a Clinical Dementia Rating (CDR) score of zero were assigned to the HC group if they had no family history of AD, or to the FHAD group if they had a parent or a sibling with AD. ADNI participants with a MMSE score from 20 to 26, impairments on the delayed recall of 1 paragraph from the Logical Memory II subscale of the Wechsler Memory Scale–Revised, a CDR score ≥ 0.5 and who met NINCDS/ADRDA criteria for probable AD (McKhann, 1984) were assigned to the AD group.

Deformation-based morphometry (DBM) processing

DBM maps were generated by concatenating the non-linear warps that mapped the T1-weighted images from each time point to the corresponding subject-specific template, and then by mapping the subject-specific template to the MNI152-2009c template. The determinant of the Jacobian matrix was used to estimate local tissue volume changes. The maps were smoothed with a 2 mm Gaussian kernel to decrease spatial noise. Finally, the natural logarithm of the Jacobian determinant was calculated. Relative to the MNI template, a value of zero indicates no volume difference, negative values indicate tissue expansion and positive values indicate tissue loss (atrophy).

Positron Emission Topography Imaging (PET) acquisition: PREVENT-AD

PET scans were performed at the McConnell Brain Imaging Centre at the MNI (Montreal, Canada) using a dedicated PET Siemens/CT high-resolution research tomograph. Tau scans were performed 80 to 100 min after radiotracer injection (9.9 ± 1.0 mCi) and Aβ scans were performed 40 to 70 min after injection (6.6 ± 0.4 mCi). T1-weighted MRI scans were acquired up to one year before the PET scans (mean interval: 8.9 ± 4.8 months) on a 3T Siemens Trio scanner at the Brain Imaging Centre of the Douglas Mental Health University Institute (Montreal, Canada). The following parameters were used: TR: 2300 ms, TE: 2.98 ms, FA: 9°; matrix size: 256 x 256; voxel size: 1 mm^3; 160-170 slices.

PET acquisition: ADNI

For flortaucipir, six five-minute frames were acquired starting 75 minutes following radiotracer injection (10.0 ± 1.0 mCi). For florbetapir, four five-minute frames were acquired starting at 50 minutes following radiotracer injection (10.0 ± 1.0 mCi).

PET processing: ADNI and PREVENT-AD

Briefly, for each participant, the PET image frames were realigned, averaged, and registered to the corresponding T1-weighted MRI processed using FreeSurfer v.6.0. Registered PET images were then masked to exclude CSF signal and finally smoothed (Sperling et al., 2011). For the smoothing, a 6 mm Gaussian kernel was used for PREVENT-AD scans, while a 8 mm kernel was applied for ADNI scans to match the approximate resolution of the lowest resolution scanners used (Jagust et al., 2015).

Sperling, R. A., Aisen, P. S., Beckett, L. A., Bennett, D. A., Craft, S., Fagan, A. M., Iwatsubo, T., Jack, C. R., Kaye, J., Montine, T. J., Park, D. C., Reiman, E. M., Rowe, C. C., Siemers, E., Stern, Y., Yaffe, K., Carrillo, M. C., Thies, B., Morrison-Bogorad, M., … Phelps, C. H. (2011). Toward defining the preclinical stages of Alzheimer’s disease: Recommendations from the National Institute on Aging-Alzheimer’s Association workgroups on diagnostic guidelines for Alzheimer’s disease. *Alzheimer’s and Dementia*, *7*(3), 280–292. https://doi.org/10.1016/j.jalz.2011.03.003

Jagust, W. J., Landau, S. M., Koeppe, R. A., Reiman, E. M., Chen, K., Mathis, C. A., Price, J. C., Foster, N. L., & Wang, A. Y. (2015). The ADNI PET Core: 2015. *Alzheimer’s & Dementia*, *11*(7), 757–771. https://doi.org/10.1016/j.jalz.2015.05.001

Diffusion-weighted MRI (DWI) acquisition: PREVENT-AD

The DWI consisted of one b_0 image and 64 diffusion-weighted volumes acquired with a b-value of 1000 s/mm^2 for all subjects. The PREVENT-AD sequence parameters were as follows: Manufacturer = SIEMENS, repetition time (TR) = 9300 ms, echo time (TE) = 92 ms, and voxel size = 2 mm.

DWI acquisition: ADNI

All axial DWI data were acquired with an echo-planar imaging sequence. The scan parameters were as follows: Manufacturer = GE MEDICAL SYSTEMS (N=69), Philips Medical Systems (N=7), SIEMENS (N=40); b-value = 583 to 1225 s/mm^2; gradient directions = 30 (N=8), 32 (N=6), 41 (N=49), 48 (N=14), 54 (N=22), 126 (N=10); voxel size = 0.91 × 0.91 mm^2 (N=20), 1.37 × 1.37 mm^2 (N=49), 2 × 2 mm^2 (N=44), 2.7 × 2.7 mm^2 (N=3); TR = 3400 to 16700 ms; TE = 55 to 105 ms; slice thickness = 2.0 mm (N=67) and 2.7 mm (N=49).

DWI processing: Tractography (Tractoflow-ABS)

The DWI processing included denoising, topup corrections, eddy-currents correction and N4 bias correction followed by the computation of DWI metrics (Theaud et al., 2020b). The following parameters were selected for diffusion image processing: DTI shells: 0 500 1000 2000; fODF shells: 0 500 1000 2000; FRF value: 10, 3, 3; algorithm: local probabilistic tracking; local seeding mask type: WM/GM interface; number of seeds per voxel: 20; spherical harmonic (SH) order: 6 (<32 gradient directions) and 8 (>=32 directions). A higher SH order allows for more complex diffusion patterns to be represented with a larger number of gradient directions (Schilling et al., 2017).

Theaud, G., Houde, J. C., Boré, A., Rheault, F., Morency, F., & Descoteaux, M. (2020b). TractoFlow: A robust, efficient and reproducible diffusion MRI pipeline leveraging Nextflow & Singularity. *NeuroImage*, *218*(April). https://doi.org/10.1016/j.neuroimage.2020.116889

Schilling, K. G., Nath, V., Blaber, J., Harrigan, R. L., Ding, Z., Anderson, A. W., & Landman, B. A. (2017). Effects of b-value and number of gradient directions on diffusion MRI measures obtained with Q-ball imaging. *Medical Imaging 2017: Image Processing*, *10133*, 101330N. https://doi.org/10.1117/12.2254545

List of the contributors involved in the PREVENT-AD Research Group

Breitner, John (McGill University; Douglas Mental Health University Institute Research Centre; StoP-Alzheimer Centre, Director, Investigator, Project Administration); Baillet, Sylvain (McGill University; Montreal Neurological Institute and Hospital, Investigator); Bellec, Pierre (Université de Montréal; Centre de recherche Institut Universitaire de Gériatrie de Montréal; McGill University; Douglas Mental Health University Institute Research Centre; StoP-Alzheimer Centre, Investigator); Bohbot, Véronique (McGill University; Douglas Mental Health University Institute Research Centre; StoP-Alzheimer Centre, Investigator); Chakravarty, Mallar (McGill University; Douglas Mental Health University Institute Research Centre; StoP-Alzheimer Centre, Investigator); Collins, D. Louis (McGill University; Montreal Neurological Institute and Hospital; Douglas Mental Health University Institute Research Centre; StoP-Alzheimer Centre, Investigator); Etienne, Pierre (McGill University; Douglas Mental Health University Institute Research Centre; StoP-Alzheimer Centre, Investigator); Evans, Alan (McGill University; Montreal Neurological Institute and Hospital; Douglas Mental Health University Institute Research Centre; StoP-Alzheimer Centre, Investigator); Gauthier, Serge (McGill University; Douglas Mental Health University Institute Research Centre; McGill University Research Centre for Studies in Aging; StoP-Alzheimer Centre, Investigator); Hoge, Rick (McGill University; Montreal Neurological Institute and Hospital; Douglas Mental Health University Institute Research Centre; StoP-Alzheimer Centre, Investigator); Ituria-Medina, Yasser (McGill University; Douglas Mental Health University Institute Research Centre; StoP-Alzheimer Centre; Montreal Neurological Institute and Hospital, Investigator); Multhaup, Gerhard (McGill University, Investigator); Münter, Lisa-Marie (McGill University, Investigator); Nair, Vasavan (McGill University; Douglas Mental Health University Institute Research Centre; McGill University Research Centre for Studies in Aging, Investigator); Poirier, Judes (McGill University; Douglas Mental Health University Institute Research Centre; StoP-Alzheimer Centre, Investigator); Rajah, Natasha (McGill University; Douglas Mental Health University Institute Research Centre; StoP-Alzheimer Centre, Investigator); Rosa-Neto, Pedro (McGill University; Douglas Mental Health University Institute Research Centre; McGill University Research Centre for Studies in Aging; StoP-Alzheimer Centre, Investigator); Soucy, Jean-Paul (McGill University; Montreal Neurological Institute and Hospital; Douglas Mental Health University Institute Research Centre; StoP-Alzheimer Centre, Investigator); Vachon-Presseau, Etienne (McGill University; Douglas Mental Health University Institute Research Centre; Northwestern University, Investigator, Consultant); Villeneuve, Sylvia (McGill University; Douglas Mental Health University Institute Research Centre; StoP-Alzheimer Centre, Investigator); Amouyel, Philippe (Université de Lille, Investigator); Appleby, Melissa (McGill University; Douglas Mental Health University Institute Research Centre; StoP-Alzheimer Centre, Investigator); Ashton, Nicholas (University of Gothenburg, Investigator); Ayranci, Gülebru (McGill University; Douglas Mental Health University Institute Research Centre; StoP-Alzheimer Centre, Investigator); Bedetti, Christophe (McGill University; Douglas Mental Health University Institute Research Centre, Investigator); Brandt, Jason (Johns Hopkins University, Investigator); Brinkmalm

Westman, Ann (University of Gothenburg, Investigator); Cuello, Claudio (McGill University; Douglas Mental Health University Institute Research Centre, Investigator); Dadar, Mahsa (McGill University; Montreal Neurological Institute and Hospital, Data Analysis); Daoust, Leslie-Ann (McGill University; Douglas Mental Health University Institute Research Centre; StoP-Alzheimer Centre, Investigator); Das, Samir (McGill University; Montreal Neurological Institute and Hospital, Investigator); Dauar-Tedeschi, Marina (McGill University; Douglas Mental Health University Institute Research Centre; McGill University Research Centre for Studies in Aging; StoP-Alzheimer Centre, Investigator); De Beaumont, Louis (Université de Montréal, Investigator); Dea, Doris (McGill University; Douglas Mental Health University Institute Research Centre; StoP-Alzheimer Centre, Investigator); Descoteaux, Maxime (Université de Sherbrooke, Consultant); Dufour, Marianne (McGill University; Douglas Mental Health University Institute Research Centre; StoP-Alzheimer Centre, Investigator); Farzin, Sarah (McGill University; Douglas Mental Health University Institute Research Centre, Investigator); Ferdinand, Fabiola (McGill University; Douglas Mental Health University Institute Research Centre; StoP-Alzheimer Centre, Investigator); Fonov, Vladimir (McGill University; Montreal Neurological Institute and Hospital, Data Analysis); Fontaine, David (McGill University; Douglas Mental Health University Institute Research Centre; StoP-Alzheimer Centre, Investigator); Gagné, Guylaine (McGill University; Douglas Mental Health University Institute Research Centre; StoP-Alzheimer Centre, Investigator); Gonneaud, Julie (McGill University; Douglas Mental Health University Institute Research Centre; StoP-Alzheimer Centre, Investigator); Kat, Justin (McGill University; Montreal Neurological Institute and Hospital; Douglas Mental Health University Institute Research Centre; StoP-Alzheimer Centre, Database Management, Database Programming); Kazazian, Christina (McGill University; Douglas Mental Health University Institute Research Centre; StoP-Alzheimer Centre, Investigator); Labonté, Anne (McGill University; Douglas Mental Health University Institute Research Centre; StoP-Alzheimer Centre, Investigator); Lafaille-Magnan, Marie-Elyse (McGill University; Douglas Mental Health University Institute Research Centre; StoP-Alzheimer Centre, Investigator); Lalancette, Marc (McGill University; Montreal Neurological Institute and Hospital, Investigator); Lambert, Jean-Charles (Université de Lille, Investigator); Leoutsakos, Jeannie-Marie (Johns Hopkins University, Investigator); Lepage, Claude (McGill University; Montreal Neurological Institute and Hospital, Investigator); Madjar, Cécile (McGill University; Montreal Neurological Institute and Hospital; Douglas Mental Health University Institute Research Centre; StoP-Alzheimer Centre, Investigator); Maillet, David (McGill University; Douglas Mental Health University Institute Research Centre, Investigator); Maltais, Jean-Robert (McGill University; Douglas Mental Health University Institute Research Centre; StoP-Alzheimer Centre; McGill University Research Centre for Studies in Aging, Investigator); Mathotaarachchi, Sulantha (McGill University; Douglas Mental Health University Institute Research Centre; McGill University Research Centre for Studies in Aging, Investigator); Mayrand, Ginette (McGill University; Douglas Mental Health University Institute Research Centre; StoP-Alzheimer Centre, Investigator); Michaud, Diane (McGill University; Douglas Mental Health University Institute Research Centre, Investigator); Montine, Thomas (Washington University,

Investigator); Morris, John (Washington University in St. Louis, Investigator); Pagé, Véronique (McGill University; Douglas Mental Health University Institute Research Centre, Investigator); Pascoal, Tharick (McGill University; Douglas Mental Health University Institute Research Centre; McGill University Research Centre for Studies in Aging; StoP-Alzheimer Centre, Investigator); Peillieux, Sandra (McGill University; Douglas Mental Health University Institute Research Centre, Investigator); Petkova, Mirela (McGill University; Montreal Neurological Institute and Hospital; Douglas Mental Health University Institute Research Centre; StoP-Alzheimer Centre, Investigator); Rioux, Pierre (McGill University; Montreal Neurological Institute and Hospital, Investigator); Sager, Mark (University of Wisconsin-Madison; School of Medicine and Public Health; Wisconsin Alzheimer Institute, Investigator); Saint-Fort, Eunice Farah (McGill University; Douglas Mental Health University Institute Research Centre, Investigator); Savard, Mélissa (McGill University; Douglas Mental Health University Institute Research Centre; StoP-Alzheimer Centre, Investigator); Sperling, Reisa (Harvard Medical School, Consultant); Tabrizi, Shirin (McGill University; Douglas Mental Health University Institute Research Centre; StoP-Alzheimer Centre, Investigator); Tariot, Pierre (Banner Alzheimer Institute, Consultant); Teigner, Eduard (McGill University; Douglas Mental Health University Institute Research Centre; StoP-Alzheimer Centre, Investigator); Thomas, Ronald (University of California San Diego; School of Medicine, Consultant); Toussaint, Paule-Joanne (McGill University; Montreal Neurological Institute and Hospital, Investigator); Tuwaig, Miranda (McGill University; Douglas Mental Health University Institute Research Centre; StoP-Alzheimer Centre, Investigator); Venugopalan, Vinod (McGill University; Douglas Mental Health University Institute Research Centre; StoP-Alzheimer Centre, Investigator); Verfaillie, Sander (Vrije Universiteit Amsterdam; Alzheimer Center; McGill University; Douglas Mental Health University Institute Research Centre, Investigator); Vogel, Jacob (McGill University; Montreal Neurological Institute and Hospital, Investigator); Wan, Karen (McGill University; Douglas Mental Health University Institute Research Centre; StoP-Alzheimer Centre, Investigator); Wang, Seqian (McGill University; Douglas Mental Health University Institute Research Centre; McGill University Research Centre for Studies in Aging, Investigator); Yu, Elsa (McGill University; Douglas Mental Health University Institute Research Centre, Investigator).

List of the contributors involved in the Alzheimer's Disease Neuroimaging Initiative (ADNI)

Michael Weiner, MD (University of California, San Francisco, Principal Investigator of ADNI), Paul Aisen, MD (University of Southern California, ATRI PI and Director of Coordinating Center Clinical Core), Ronald Petersen, MD, PhD (Mayo Clinic, Rochester, co-PI of Clinical Core), Clifford R. Jack, Jr., MD (Mayo Clinic, Rochester, Executive Committee Member), William Jagust, MD (University of California, Berkeley, Executive Committee Member), Susan Landau, PhD (University of California, Berkeley, Executive Committee Member), Monica Rivera-Mindt, PhD (Fordham University; Mt. Sinai Medical Center, Executive Committee Member), Ozioma Okonkwo, PhD (University of Wisconsin, Executive Committee Member), Leslie M. Shaw, PhD (University of Pennsylvania, Executive Committee Member), Edward B. Lee, MD, PhD (University of Pennsylvania, Executive Committee Member), Arthur W. Toga, PhD (University of California, Los Angeles, Executive Committee Member), Laurel Beckett, PhD (University of California, Davis, Executive Committee Member), Danielle Harvey, PhD (University of California, Davis, Executive Committee Member), Robert C. Green, MD, MPH (Boston University, Executive Committee Member), Andrew J. Saykin, PsyD (Indiana University, Executive Committee Member), Kwangsik Nho, PhD (Indiana University, Executive Committee Member), Richard J. Perrin, MD, PhD (Washington University St. Louis, Executive Committee Member), Duygu Tosun, PhD (University of California, San Francisco, Executive Committee Member), Pallavi Sachdev, PhD (Eisai, Chair of ADNI4 Private Partner Scientific Board 2023-2024), Robert C. Green, MD, MPH (Harvard University, Chair of Data and Publication Committee), Erin Drake (Harvard University, Data and Publication Committee Member), Tom Montine, MD, PhD (University of Washington, Chair of Resource Allocation Review Committee), Cat Conti, BA (Northern California Institute for Research and Education, Resource Allocation Review Committee Member), Michael Rafii, MD, PhD (University of Southern California, Clinical Core Leader), Rema Raman, PhD (University of Southern California, Clinical Core Leader), Michael Donohue, PhD (University of Southern California, Clinical Core Leader), Jennifer Salazar, MBS (University of Southern California, Clinical Core Leader), Laurel Beckett, PhD (University of California, Davis, Biostatistics Core Leader), Danielle Harvey, PhD (University of California, Davis, Biostatistics Core Leader), Ozioma Okonkwo, PhD (University of Wisconsin, Engagement Core Leader), Monica Rivera-Mindt, PhD (Fordham University; Mt. Sinai, Engagement Core Leader), Clifford R. Jack, Jr., MD (Mayo Clinic, Rochester, MRI Core Leader), Nick C. Fox, MD (University College London, MRI Core Leader), Paul Thompson, PhD (UCLA School of Medicine, MRI Core Leader), Charles DeCarli, MD (University of California, Davis, MRI Core Leader), William Jagust, MD (University of California, Berkeley, PET Core Leader), Susan Landau, PhD (University of California, Berkeley, PET Core Leader), Robert A. Koeppe, PhD (University of Michigan, PET Core Leader), Richard J. Perrin, MD, PhD (Washington University St. Louis, Neuropathology Core Leader), Leslie Shaw, PhD (University of Pennsylvania, Biomarkers Core Leader), Edward B. Lee, MD, PhD (University of Pennsylvania, Biomarkers Core Leader), Virginia M.Y. Lee, PhD, MBA (University of Pennsylvania, Biomarkers Core Leader), Arthur W. Toga, PhD (University of Southern California, Informatics

Core Leader), Andrew J. Saykin, PsyD (Indiana University School of Medicine, Genetics Core Leader), Kwangsik Nho, PhD (Indiana University School of Medicine, Genetics Core Leader), Rima Kaddurah-Daouk, PhD (Duke University/AD Metabolomics Consortium, Genetics Core Leader), Jason Karlawish, MD (University of Pennsylvania, ADNI4 Amyloid Disclosure Team), Michael W. Weiner, MD (UCSF/NCIRE, Early Project Development), Neil Buckholtz, PhD (National Institute on Aging, NIA Representative), Laurie Ryan, PhD (National Institute on Aging, NIA Representative), Zaven Khachaturian, PhD (Prevent Alzheimer's Disease 2020, Chair of ADNI External Scientific Advisory Board), Maria Carrillo, PhD (Alzheimer's Association, ADNI External Scientific Advisory Board Member), William Potter, MD (National Institute of Mental Health, ADNI External Scientific Advisory Board Member), Eliezer Masliah, MD (NIA, ADNI External Scientific Advisory Board Member).

Supplementary Figure 1. Regions with significantly more (A) and less (B) atrophy progression between the participants with Alzheimer's disease (AD) and individuals with a family history of Alzheimer's disease (FHAD)**. Different colors represent distinct cortical regions of the atlas.**

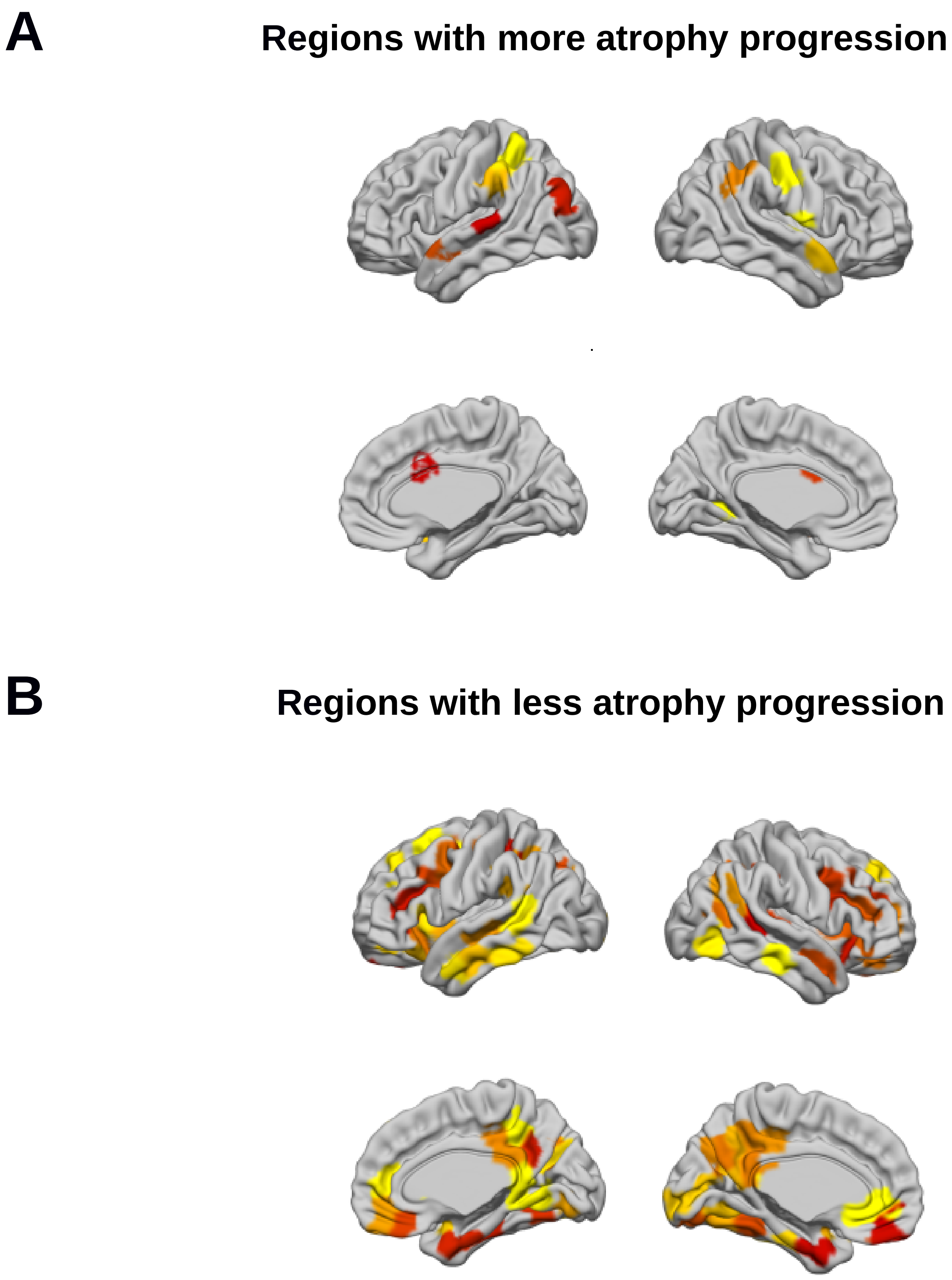

Supplementary Figure 2. Beta values associated with the group effect in cortical regions showing significantly higher tau (A) and beta-amyloide (Aβ) accumulation (B) in participants with Alzheimer's disease (AD: N_{tau}=58, $N_{Aβ}$ =145) vs. individuals with a family history of Alzheimer's disease (FHAD: N_{tau}=96, $N_{Aβ}$ =165). Linear regression models were performed with regional tau-PET (A) or Aβ-PET (B) binding (standardized uptake value ratios) as the dependent variable, group (FHAD, AD) as the independent variable, and age at baseline, sex, education, BMI, and *APOe4* status as covariates. All *p*-values were corrected for multiple comparisons (448 cortical regions) using the false discovery rate (FDR) method.

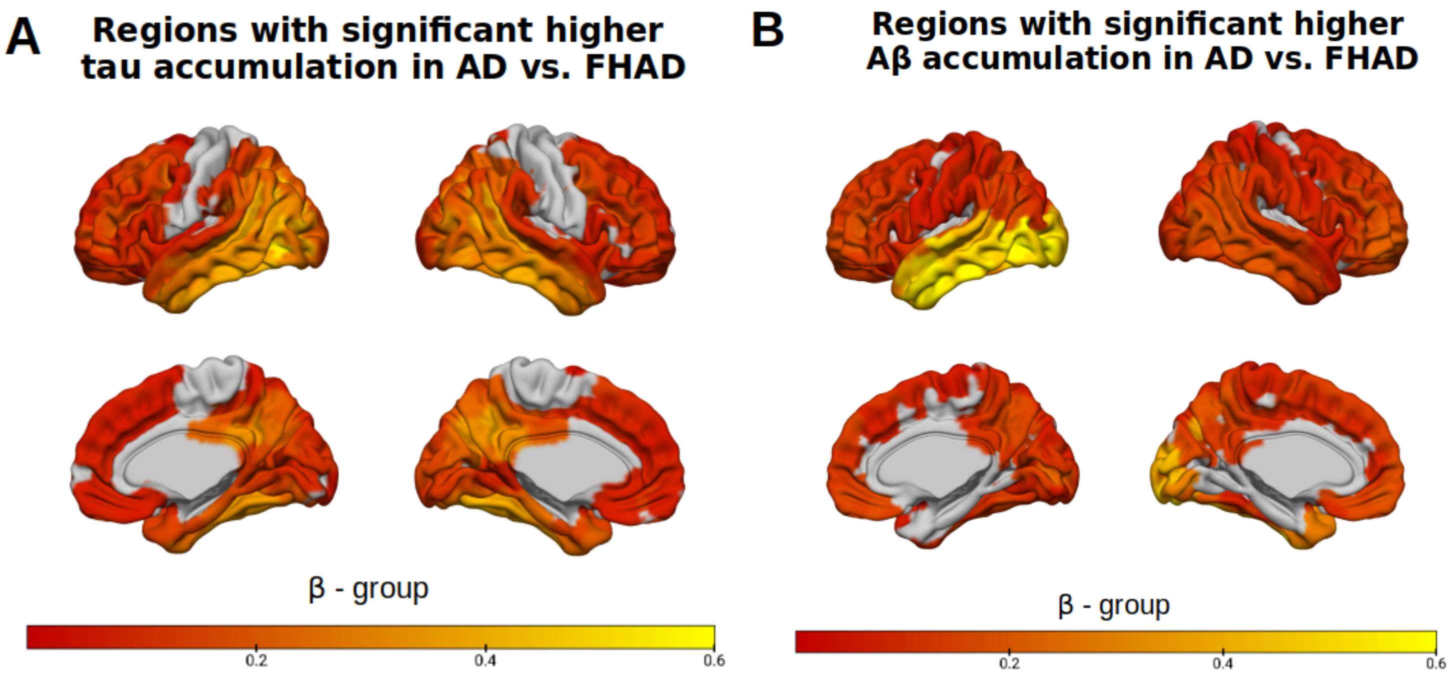

Supplementary Figure 3. Regional Pearson's correlations between atrophy at baseline, and tau (A) and Aβ accumulation (B) (N=448) in participants with Alzheimer's disease (AD: N_{tau}=14, $N_{A\beta}$ =40) and individuals with a family history of Alzheimer's disease (FHAD: N_{tau}=35, $N_{A\beta}$ =92) using only participants who had both PET and MRI data at baseline

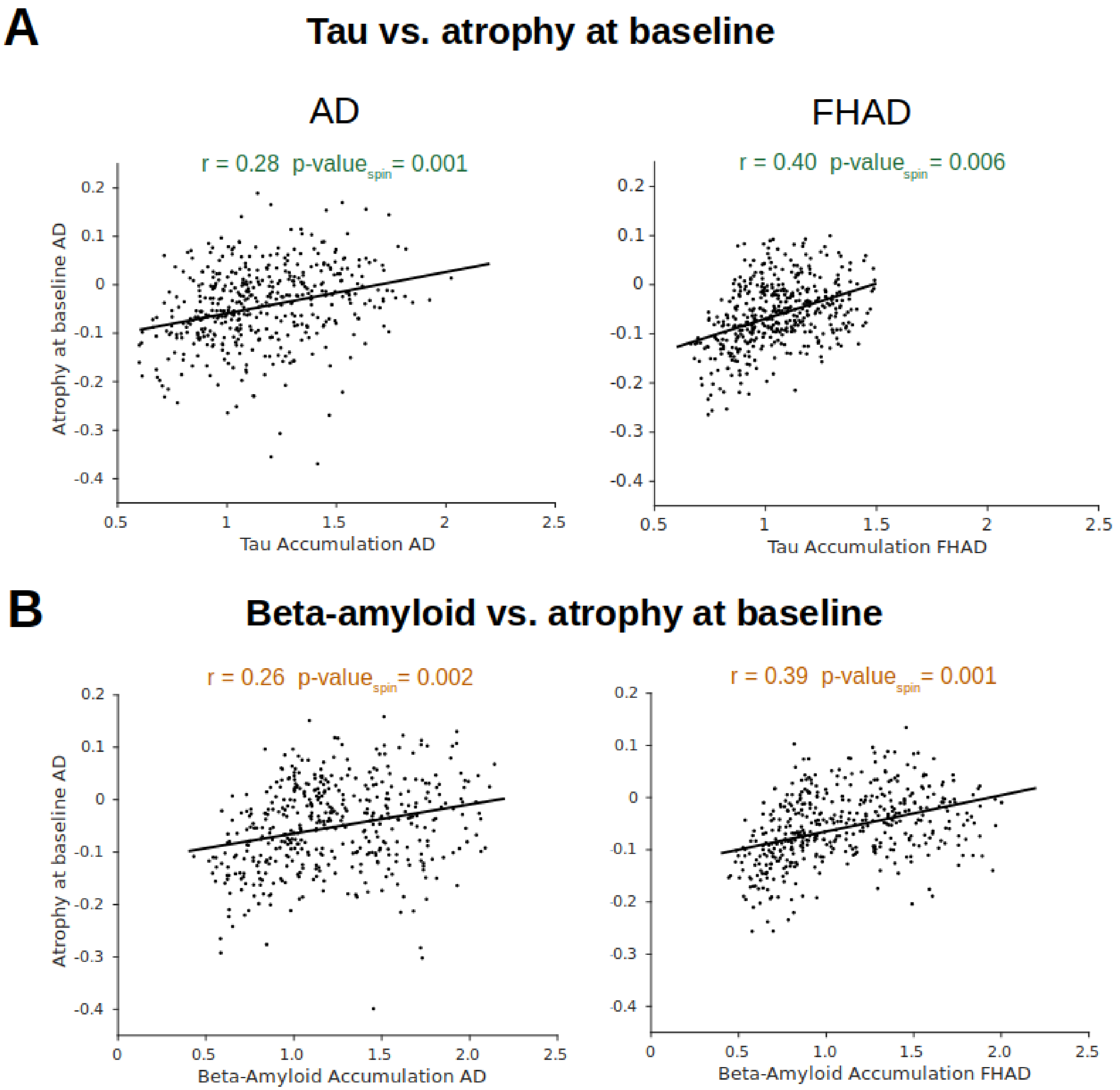

Supplementary Table 1

Number of subjects at each main step of the method for the participants with Alzheimer's disease (AD), individuals with a family history of AD (FHAD) and healthy controls (HC) in the ADNI and PREVENT-AD or MALS database separately

Data	Group	Database	Raw data ADNI	Raw data PREVENT-AD or MALS	Processing + QC ADNI	Processing + QC PREVENT-AD or MALS	Site harmonization ADNI	Group balancing ADNI	Group balancing PREVENT-AD or MALS
**ATROPHY PROGRESSION - DEFORMATION-BASED MORPHOMETRY (DBM)**									
T1w-MRI	AD	ADNI	Bl: 418 1Y: 237	NA	Bl: 340 1Y: 190	NA	No data removed	Bl: 70 1Y: 96	NA
			2Y: 153 3Y:18	NA	2Y: 121 3Y:17	NA		2Y: 55 3Y: 5	NA
	FHAD	ADNI PREVENT-AD	Bl: 141 1Y:120	Bl: 179 1Y: 128	Bl: 117 1Y: 100	Bl: 163 1Y: 116	No data removed	Bl: 108 1Y: 93	Bl: 45 1Y: 33
			2Y: 116 3Y: 58	2Y: 63 3Y: 7	2Y: 97 3Y: 46	2Y: 58 3Y: 7		2Y: 89 3Y: 44	2Y: 21 3Y: 46
			4Y: 74	4Y: 25	4Y: 61	4Y: 22		4Y: 55	4Y: 10
	HC	ADNI MALS	Bl: 105 1Y: 70	Bl: 48 1Y: 0	Bl: 85 1Y: 55	Bl: 46 1Y: 0	Bl: 81 1Y: 55	Bl: 70 1Y: 47	Bl: 46 1Y: 0
			2Y: 70 3Y: 48	2Y: 0 3Y: 0	2Y: 56 3Y: 39	2Y: 0 3Y: 0	2Y: 56 3Y: 39	2Y: 48 3Y: 33	2Y: 0 3Y: 0
			4Y: 27	4Y: 0	4Y: 20	4Y: 0	4Y: 20	4Y: 18	4Y: 0
**POSITRON EMISSION TOPOGRAPHY IMAGING (PET)**									
Tau-PET	AD	ADNI	Bl: 64	NA	Bl: 62	NA	Bl: 58	Bl: 58	NA
	FHAD	ADNI PREVENT-AD	Bl: 46	Bl: 120	Bl: 37	Bl: 120	Bl: 26	Bl: 25	Bl: 71
Aβ-PET	AD	ADNI	Bl: 167	NA	Bl: 159	NA	Bl: 154	Bl: 145	NA
	FHAD	ADNI PREVENT-AD	Bl: 99	Bl: 122	Bl: 97	Bl: 122	Bl: 92	Bl: 87	Bl: 78
**STRUCTURAL CONNECTIVITY - DIFFUSION TENSOR IMAGING (DTI)**									
DWI-MRI	AD	ADNI	Bl: 116	NA	Bl: 91	NA	No data removed	Bl: 72	NA
	FHAD	PREVENT-AD	NA	Bl: 304	NA	Bl: 277	No data removed	NA	Bl: 78

QC: Quality Control, CR: cortical regions, Bl: Baseline, Y: Year

Supplementary Table 2

Cortical regions of the Cammoun atlas with significant difference in atrophy progression between groups: group*age interaction (F-test)

Cortical regions	Hemisphere	Beta group*age	P-value$_{FDR}$
lingual_6	left	0.006	0.00002
superiorparietal_4	left	0.004	0.0000004
supramarginal_8	left	0.003	0.0001
posteriorcingulate_3	right	0.003	0.003
posteriorcingulate_2	left	0.003	0.002
caudalanteriorcingulate_3	right	0.003	0.0004
precuneus_11	left	0.002	0.02
precentral_12	right	0.002	0.002
supramarginal_5	left	0.002	0.002
pericalcarine_1	right	0.002	0.008
inferiorparietal_2	right	0.002	0.009
superiortemporal_9	left	0.002	0.003
superiortemporal_1	left	0.002	0.009
caudalanteriorcingulate_1	left	0.002	0.02
posteriorcingulate_1	right	0.002	0.008
lingual_7	right	0.002	0.03
superiortemporal_8	right	0.002	0.003
cuneus_4	right	0.002	0.01
transversetemporal_1	left	0.002	0.03
postcentral_6	right	0.002	0.009
supramarginal_3	right	0.002	0.02
precentral_10	right	0.002	0.03
postcentral_6	left	0.001	0.04
lateraloccipital_3	right	0.001	0.01
postcentral_2	right	0.001	0.02
superiortemporal_4	left	0.001	0.02
inferiorparietal_2	left	0.001	0.03
superiortemporal_8	left	0.001	0.01
postcentral_14	left	0.001	0.03
lingual_6	right	-0.001	0.03
superiorparietal_11	right	-0.001	0.03
rostralmiddlefrontal_9	left	-0.001	0.03
parsopercularis_3	left	-0.001	0.04
superiorfrontal_5	left	-0.001	0.03
fusiform_2	left	-0.001	0.049
parahippocampal_1	right	-0.001	0.03
pericalcarine_2	left	-0.001	0.03
superiortemporal_7	left	-0.001	0.04
medialorbitofrontal_1	right	-0.001	0.0497
posteriorcingulate_4	right	-0.001	0.02
superiorfrontal_3	left	-0.001	0.03
superiorfrontal_2	right	-0.001	0.02
precuneus_3	right	-0.001	0.03
posteriorcingulate_4	left	-0.001	0.02
insula_5	left	-0.001	0.03
isthmuscingulate_2	right	-0.001	0.003

insula_4	left	-0.001	0.03
rostralanteriorcingulate_1	left	-0.001	0.009
middletemporal_4	left	-0.001	0.006
superiorparietal_1	right	-0.001	0.02
lateraloccipital_10	right	-0.001	0.005
superiorfrontal_6	left	-0.001	0.01
middletemporal_7	left	-0.001	0.02
precuneus_1	right	-0.001	0.02
fusiform_6	left	-0.001	0.01
superiorfrontal_13	left	-0.001	0.03
supramarginal_9	left	-0.001	0.03
lateralorbitofrontal_6	left	-0.001	0.008
pericalcarine_1	left	-0.002	0.003
superiortemporal_1	right	-0.002	0.02
inferiorparietal_4	right	-0.002	0.02
precuneus_3	left	-0.002	0.01
superiorfrontal_13	right	-0.002	0.008
bankssts_2	left	-0.002	0.01
middletemporal_8	right	-0.002	0.02
lateralorbitofrontal_5	left	-0.002	0.008
precentral_13	right	-0.002	0.04
lingual_4	left	-0.002	0.004
middletemporal_6	right	-0.002	0.002
superiorfrontal_10	left	-0.002	0.009
supramarginal_6	left	-0.002	0.006
lingual_1	left	-0.002	0.001
fusiform_5	left	-0.002	0.004
bankssts_3	left	-0.002	0.004
superiorparietal_5	left	-0.002	0.021
parsopercularis_2	right	-0.002	0.004
precuneus_4	right	-0.002	0.008
inferiorparietal_8	left	-0.002	0.01
parahippocampal_3	right	-0.002	0.01
rostralmiddlefrontal_3	right	-0.002	0.01
middletemporal_4	right	-0.002	0.02
parsopercularis_1	left	-0.002	0.002
inferiortemporal_5	left	-0.002	0.001
insula_5	right	-0.002	0.002
precuneus_10	left	-0.002	0.0002
precuneus_6	left	-0.002	0.001
superiortemporal_7	right	-0.002	0.01
medialorbitofrontal_2	right	-0.002	0.0003
entorhinal_1	right	-0.002	0.012
fusiform_1	right	-0.002	0.006
superiorfrontal_6	right	-0.002	0.0004
inferiorparietal_10	right	-0.002	0.02
middletemporal_6	left	-0.002	0.001
inferiorparietal_1	right	-0.002	0.005
precuneus_10	right	-0.002	0.0003
parahippocampal_2	left	-0.002	0.003
isthmuscingulate_1	right	-0.002	0.001
superiorfrontal_12	left	-0.002	0.0003

caudalmiddlefrontal_6	left	-0.002	0.020
lingual_2	right	-0.002	0.0002
precuneus_7	left	-0.002	0.0002
lateralorbitofrontal_6	right	-0.002	0.0003
precuneus_8	left	-0.002	0.001
superiorfrontal_7	right	-0.002	0.0004
bankssts_1	right	-0.002	0.007
medialorbitofrontal_3	left	-0.002	0.002
precentral_7	left	-0.002	0.004
superiortemporal_6	left	-0.002	0.0005
caudalmiddlefrontal_4	right	-0.002	0.0004
inferiortemporal_4	left	-0.002	0.002
middletemporal_5	left	-0.002	0.0004
medialorbitofrontal_3	right	-0.002	0.0005
lateraloccipital_9	left	-0.002	0.001
fusiform_7	left	-0.002	0.0002
insula_6	left	-0.002	0.00008
isthmuscingulate_2	left	-0.002	0.000009
parsopercularis_4	right	-0.002	0.0007
rostralmiddlefrontal_8	right	-0.002	0.002
caudalmiddlefrontal_3	left	-0.002	0.0003
superiorparietal_3	left	-0.002	0.0008
precuneus_7	right	-0.003	0.0004
rostralmiddlefrontal_5	right	-0.003	0.002
caudalmiddlefrontal_4	left	-0.003	0.0004
precentral_11	right	-0.003	0.002
parstriangularis_1	right	-0.003	0.00002
insula_7	right	-0.003	0.00003
fusiform_1	left	-0.003	0.00006
lateralorbitofrontal_4	left	-0.003	0.000003
parahippocampal_1	left	-0.003	0.00004
supramarginal_1	right	-0.003	0.00006
lateralorbitofrontal_2	right	-0.003	0.000003
fusiform_7	right	-0.003	0.000006
rostralmiddlefrontal_1	left	-0.003	0.00002
entorhinal_1	left	-0.003	0.0003
fusiform_2	right	-0.003	0.000004
medialorbitofrontal_2	left	-0.003	0.000001
bankssts_2	right	-0.003	0.000009
parstriangularis_3	left	-0.004	0.000000009

Supplementary Table 3

Cortical regions of the Cammoun atlas with significant difference in atrophy progression in Alzheimer's disease (AD) compared with age-expected effect in HC

Cortical regions	Hemisphere	Beta group*age	P-value$_{FDR}$
lingual_6	left	0.01	0.00003
superiorparietal_4	left	0.008	0.0000002
posteriorcingulate_3	right	0.007	0.001
posteriorcingulate_2	left	0.007	0.0007
supramarginal_8	left	0.007	0.00006
caudalanteriorcingulate_3	right	0.006	0.00008
precuneus_11	left	0.006	0.008
precentral_12	right	0.005	0.0005
lingual_7	right	0.005	0.004
postcentral_6	left	0.005	0.0003
pericalcarine_1	right	0.004	0.005
caudalanteriorcingulate_1	left	0.004	0.005
superiortemporal_1	left	0.004	0.004
transversetemporal_1	left	0.004	0.007
cuneus_4	right	0.004	0.005
inferiorparietal_2	right	0.004	0.009
posteriorcingulate_1	right	0.004	0.006
superiortemporal_9	left	0.004	0.004
supramarginal_5	left	0.004	0.007
precentral_10	right	0.004	0.01
lateraloccipital_3	right	0.003	0.01
superiortemporal_8	right	0.003	0.009
superiortemporal_8	left	0.003	0.01
postcentral_14	left	0.002	0.01
lingual_1	left	-0.002	0.049
isthmuscingulate_2	right	-0.002	0.02
lateraloccipital_10	right	-0.002	0.03
rostralanteriorcingulate_1	left	-0.002	0.04
pericalcarine_1	left	-0.002	0.03
lateralorbitofrontal_6	left	-0.002	0.03
lateralorbitofrontal_5	left	-0.003	0.04
lingual_4	left	-0.003	0.04
parsopercularis_3	left	-0.003	0.02
superiorfrontal_5	left	-0.003	0.01
superiorfrontal_6	left	-0.003	0.02
middletemporal_4	left	-0.003	0.01
superiortemporal_7	left	-0.003	0.03
bankssts_2	left	-0.003	0.03
fusiform_6	left	-0.003	0.01
precuneus_10	left	-0.003	0.003
superiorfrontal_10	left	-0.003	0.02
precuneus_6	left	-0.003	0.009
precuneus_3	right	-0.003	0.01
rostralmiddlefrontal_9	left	-0.003	0.002
precuneus_7	left	-0.003	0.004
isthmuscingulate_1	right	-0.003	0.007

caudalmiddlefrontal_4	right	-0.003	0.01
medialorbitofrontal_2	right	-0.003	0.002
parahippocampal_1	right	-0.003	0.005
middletemporal_7	left	-0.003	0.009
middletemporal_6	right	-0.003	0.004
superiorfrontal_13	right	-0.003	0.008
lateralorbitofrontal_6	right	-0.003	0.003
parsopercularis_1	left	-0.003	0.007
precuneus_8	left	-0.003	0.01
precentral_7	left	-0.003	0.03
bankssts_3	left	-0.003	0.006
middletemporal_4	right	-0.003	0.03
lingual_2	right	-0.003	0.002
insula_5	right	-0.003	0.002
parahippocampal_3	right	-0.003	0.01
inferiortemporal_5	left	-0.003	0.002
superiortemporal_6	left	-0.004	0.003
parsopercularis_2	right	-0.004	0.002
middletemporal_8	right	-0.004	0.007
parsopercularis_4	right	-0.004	0.01
middletemporal_6	left	-0.004	0.002
superiorparietal_3	left	-0.004	0.02
medialorbitofrontal_3	right	-0.004	0.002
bankssts_1	right	-0.004	0.009
superiorfrontal_7	right	-0.004	0.001
precuneus_7	right	-0.004	0.008
isthmuscingulate_2	left	-0.004	0.0001
parahippocampal_2	left	-0.004	0.002
fusiform_1	right	-0.004	0.003
superiorfrontal_12	left	-0.004	0.0001
precuneus_10	right	-0.004	0.0001
superiorfrontal_6	right	-0.004	0.0001
middletemporal_5	left	-0.004	0.0004
caudalmiddlefrontal_3	left	-0.004	0.002
rostralmiddlefrontal_5	right	-0.004	0.01
precentral_11	right	-0.004	0.008
inferiortemporal_4	left	-0.004	0.0006
rostralmiddlefrontal_8	right	-0.005	0.004
insula_6	left	-0.005	0.00002
medialorbitofrontal_2	left	-0.005	0.0004
lateraloccipital_9	left	-0.005	0.0004
entorhinal_1	left	-0.005	0.002
rostralmiddlefrontal_1	left	-0.005	0.0004
parstriangularis_1	right	-0.005	0.00006
caudalmiddlefrontal_4	left	-0.005	0.0009
lateralorbitofrontal_2	right	-0.005	0.00004
fusiform_7	left	-0.005	0.00001
insula_7	right	-0.005	0.00003
lateralorbitofrontal_4	left	-0.005	0.000003
fusiform_7	right	-0.005	0.00002
supramarginal_1	right	-0.005	0.0002
fusiform_1	left	-0.005	0.00005

bankssts_2	right	-0.005	0.0005
fusiform_2	right	-0.006	0.00001
parahippocampal_1	left	-0.006	0.000003
parstriangularis_3	left	-0.007	0.00000001

Supplementary Table 4

Cortical regions of the Cammoun atlas with significant difference in atrophy progression in individuals with a family history of Alzheimer's disease (FHAD) compared with age-expected effect in HC

Cortical regions	Hemisphere	Beta group*age	P-value$_{FDR}$
postcentral_6	left	0.006	0.0003
posteriorcingulate_3	right	0.005	0.04
lingual_7	right	0.004	0.01
posteriorcingulate_2	left	0.004	0.04
caudalanteriorcingulate_3	right	0.004	0.01
superiorparietal_4	left	0.004	0.02
precentral_12	right	0.004	0.02
transversetemporal_1	left	0.003	0.04
caudalanteriorcingulate_1	left	0.003	0.047
cuneus_4	right	0.003	0.0497
superiorfrontal_12	left	-0.002	0.04
lateralorbitofrontal_4	left	-0.002	0.049
precuneus_10	right	-0.003	0.02
insula_6	left	-0.003	0.03
fusiform_1	left	-0.003	0.0495
parahippocampal_1	right	-0.003	0.02
superiorfrontal_6	right	-0.003	0.01
rostralmiddlefrontal_9	left	-0.003	0.005
lateraloccipital_9	left	-0.003	0.03
parstriangularis_3	left	-0.003	0.02
fusiform_7	left	-0.004	0.004
parahippocampal_1	left	-0.004	0.007

Supplementary Table 5

Pearson's correlations with structurally and non-structurally connected neighbors' atrophy and PET concentration using structural connectivity from healthy adults

Measure	Structurally connected neighbors		Non-structurally connected neighbors	
	Pearson's r	p-value spin test	Pearson's r	p-value spin test
**Group with a family history of Alzheimer's disease (FHAD)**				
**Atrophy progression (beta)**	**0.36**	**0.02**	**-0.36**	**0.001**
**Atrophy at baseline**	**0.44**	**0.001**	**-0.50**	**0.001**
**Tau-PET baseline**	**0.24**	**0.009**	**-0.34**	**0.001**
**Beta-Amyloid-PET baseline**	**0.29**	**0.003**	**-0.39**	**0.001**
**Group with Alzheimer's disease (AD)**				
**Atrophy progression (beta)**	**0.33**	**0.02**	**-0.43**	**0.001**
**Atrophy at baseline**	**0.66**	**0.001**	**-0.67**	**0.001**
**Tau-PET baseline**	**0.49**	**0.001**	**-0.36**	**0.001**
**Beta-Amyloid-PET baseline**	**0.21**	**0.004**	**-0.32**	**0.001**

Supplementary Table 6

Pearson's correlations with structurally and non-structurally connected neighbors' atrophy or PET concentration using group-specific structural connectivity

Measure	Structurally connected neighbors		Non-structurally connected neighbors	
	Pearson's r	p-value spin test	Pearson's r	p-value spin test
**Group with a family history of Alzheimer's disease (FHAD)**				
**Atrophy progression (beta)**	**0.31**	**0.03**	**-0.36**	**0.02**
**Atrophy at baseline**	**0.26**	**0.04**	**-0.35**	**0.001**
**Tau-PET baseline**	**0.29**	**0.001**	**-0.39**	**0.001**
**Beta-Amyloid-PET baseline**	**0.30**	**0.006**	**-0.39**	**0.001**
**Group with Alzheimer's disease (AD)**				
**Atrophy progression (beta)**	0.11	0.26	-0.16	0.18
**Atrophy at baseline**	**0.50**	**0.001**	**-0.49**	**0.001**
**Tau-PET baseline**	**0.41**	**0.03**	**-0.44**	**0.02**
**Beta-Amyloid-PET baseline**	**0.28**	**0.02**	**-0.32**	**0.01**

Supplementary Table 7

Pearson's correlations between atrophy progression and different neurotransmitter receptor and transporter distributions

	Pearson's r	p-value spin test	p-value spin-FDR
**Group with a family history of Alzheimer's disease (FHAD)**			
Norepinephrine (NET1)	0.04	0.41	0.47
Serotonine (5-HT1B)	-0.02	0.41	0.47
Serotonine (5-HT6)	-0.15	0.04	0.19
Acetycholine (VachT)	0.09	0.24	0.40
Glutamate (mGluR5)	-0.18	0.09	0.25
Dopamine (D2)	-0.19	0.15	0.30
Histamine (H3)	-0.08	0.33	0.44
**Group with Alzheimer's disease (AD)**			
Norepinephrine (NET1)	0.10	0.22	0.39
Serotonine (5-HT1B)	0.02	0.42	0.47
Serotonine (5-HT6)	**-0.22**	**0.004**	**0.04**
Acetycholine (VachT)	0.008	0.49	0.49
Glutamate (mGluR5)	-0.16	0.12	0.26
Dopamine (D2)	-0.21	0.12	0.26
Histamine (H3)	0.09	0.29	0.43

Supplementary Table 8

Pearson's correlations between atrophy at baseline (corrected for site effect) and, neurotransmitter receptor and transporter distributions

Receptor/transporter	Correlation between atrophy at baseline and neurotransmitter receptor/transporter distributions		
	Pearson's r	p-value spin test	p-value spin-FDR
**Group with Alzheimer's disease (AD)**			
Norepinephrine (NET1)	0.04	0.40	0.40
Serotonine (5-HT1B)	0.15	0.12	0.17
Serotonine (5-HT6)	**0.35**	**0.001**	**0.004**
Acetycholine (VachT)	-0.05	0.36	0.40
Glutamate (mGluR5)	**0.42**	**0.001**	**0.004**
Dopamine (D2)	0.20	0.17	0.22
Histamine (H3)	0.14	0.23	0.28
**Group with a family history of Alzheimer's disease (FHAD)**			
Norepinephrine (NET1)	0.25	0.046	0.08
Serotonine (5-HT1B)	**0.32**	**0.004**	**0.01**
Serotonine (5-HT6)	**0.46**	**0.001**	**0.004**
Acetycholine (VachT)	0.22	0.047	0.08
Glutamate (mGluR5)	**0.48**	**0.001**	**0.004**
Dopamine (D2)	0.08	0.31	0.36
Histamine (H3)	0.33	0.03	0.06
**Group without FHAD (HC)**			
Norepinephrine (NET1)	0.26	0.049	0.08
Serotonine (5-HT1B)	**0.33**	**0.003**	**0.01**
Serotonine (5-HT6)	**0.45**	**0.001**	**0.004**
Acetycholine (VachT)	0.21	0.06	0.09
Glutamate (mGluR5)	**0.44**	**0.001**	**0.004**
Dopamine (D2)	0.05	0.40	0.40
Histamine (H3)	0.34	0.03	0.06
